# Coupling gene regulatory patterns to bioprocess conditions to optimize synthetic metabolic modules for improved sesquiterpene production in yeast

**DOI:** 10.1186/s13068-017-0728-x

**Published:** 2017-02-21

**Authors:** Bingyin Peng, Manuel R. Plan, Alexander Carpenter, Lars K. Nielsen, Claudia E. Vickers

**Affiliations:** 1 Australian Institute for Bioengineering and Nanotechnology (AIBN), The University of Queensland, St. Lucia, QLD 4072 Australia; 2 Metabolomics Australia (Queensland Node), The University of Queensland, St. Lucia, QLD 4072 Australia

**Keywords:** *Saccharomyces cerevisiae*, Sesquiterpene, Synthetic biology, Metabolic engineering, Microbial cell factories, Transcription regulation, Mevalonate pathway, Fed-batch cultivation, Overflow metabolism

## Abstract

**Background:**

Assembly of heterologous metabolic pathways is commonly required to generate microbial cell factories for industrial production of both commodity chemicals (including biofuels) and high-value chemicals. Promoter-mediated transcriptional regulation coordinates the expression of the individual components of these heterologous pathways. Expression patterns vary during culture as conditions change, and this can influence yeast physiology and productivity in both positive and negative ways. Well-characterized strategies are required for matching transcriptional regulation with desired output across changing culture conditions.

**Results:**

Here, constitutive and inducible regulatory mechanisms were examined to optimize synthetic isoprenoid metabolic pathway modules for production of *trans*-nerolidol, an acyclic sesquiterpene alcohol, in yeast. The choice of regulatory system significantly affected physiological features (growth and productivity) over batch cultivation. Use of constitutive promoters resulted in poor growth during the exponential phase. Delaying expression of the assembled metabolic modules using the copper-inducible *CUP1* promoter resulted in a 1.6-fold increase in the exponential-phase growth rate and a twofold increase in productivity in the post-exponential phase. However, repeated use of the *CUP1* promoter in multiple expression cassettes resulted in genetic instability. A diauxie-inducible expression system, based on an engineered *GAL* regulatory circuit and a set of four different *GAL* promoters, was characterized and employed to assemble nerolidol synthetic metabolic modules. Nerolidol production was further improved by 60% to 392 mg L^−1^ using this approach. Various carbon source systems were investigated in batch/fed-batch cultivation to regulate induction through the *GAL* system; final nerolidol titres of 4–5.5 g L^−1^ were achieved, depending on the conditions.

**Conclusion:**

Direct comparison of different transcriptional regulatory mechanisms clearly demonstrated that coupling the output strength to the fermentation stage is important to optimize the growth fitness and overall productivities of engineered cells in industrially relevant processes. Applying different well-characterized promoters with the same induction behaviour mitigates against the risks of homologous sequence-mediated genetic instability. Using these approaches, we significantly improved sesquiterpene production in yeast.

**Electronic supplementary material:**

The online version of this article (doi:10.1186/s13068-017-0728-x) contains supplementary material, which is available to authorized users.

## Background

Metabolic engineering and synthetic biology are now routinely used for the engineering of microorganisms for industrial production of desirable chemicals, including fuels and biochemicals [1–3]. In the first instance, metabolic pathway flux towards the desired product is optimized by introduction of enzymes with the best catalytic efficiency (which are often heterologous) [4–6]. Expression levels of these enzymes are then titrated for optimal pathway balance, in combination with other metabolic engineering strategies to redirect carbon in the metabolic network [7–9]. Complicating matters, the activities of synthetic pathways should be coordinated with the dynamic fermentation conditions and process stage [10]. Imbalance in pathway flux can have a negative effect on cell physiology (e.g. growth rate) and on product titre [11]. Coordination can be controlled at the transcriptional level to regulate gene expression (and hence, enzyme activity). However, there is only limited information available on the dynamic behaviour of promoters across the range of conditions that occur in industrial fermentation processes.

**Fig. 1:**
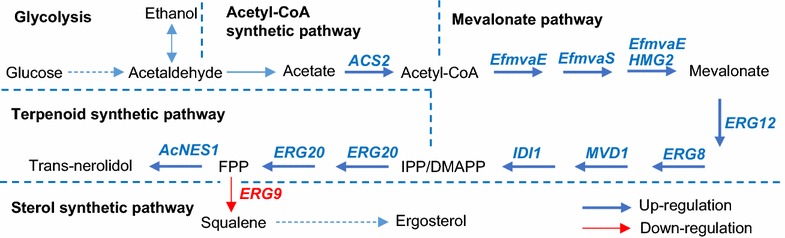
Metabolic pathways for trans-nerolidol (sesquiterpene) production in yeast. *ACS2* acetyl-CoA synthase, *EfmvaE Enterococcus faecalis* acetoacetyl-CoA thiolase/HMG-CoA reductase, *EfmvaS E. faecalis* HMG-CoA synthase, *HMG2* HMG-CoA reductase 2, *ERG12* mevalonate kinase, *ERG8* phosphomevalonate kinase, *MVD1* mevalonate pyrophosphate decarboxylase, *IDI1* isopentenyl diphosphate:dimethylallyl diphosphate isomerase, *ERG20* farnesyl pyrophosphate synthetase, *AcNES1 Actinidia chinensis* tran-nerolidol synthase, *ERG9* squalene synthase, *IPP* isopentenyl pyrophosphate, *DMAPP* dimethylallyl pyrophosphate, *FPP* farnesyl pyrophosphate. *Dashed arrow* means multiple biochemical steps

The budding yeast *Saccharomyces cerevisiae* is a common engineering platform for production of high-value plant terpenoids. Terpenoids are a diverse class of chemicals naturally synthesized from the universal 5-carbon precursors, isopentenyl pyrophosphate (IPP) and dimethylallyl pyrophosphate (DMAPP) [12]. The sesquiterpene sub-class of terpenes have 15 carbon atoms and are synthesized from farnesyl pyrophosphate (FPP), which is condensed from one molecule of DMAPP and two molecules of IPP. Sesquiterpenes have broad industrial applications, including as fragrances, flavours, pharmaceuticals, solvents and fuels. A generic set of metabolic engineering approaches can be used to improve sesquiterpene production (Fig. 1) [13–16]. First, high-level production of terpenoids requires improved flux from central carbon metabolism to the sesquiterpene precursor FPP [16–23]. This is typically achieved by augmenting the mevalonate (MVA) pathway through overexpression or heterologous expression of individual genes (including farnesyl pyrophosphate synthase, FPPS). Enhanced MVA pathway activity causes squalene accumulation [23–25] and it is necessary to constrain the flux-competing squalene synthase to redirect FPP flux away from sterol production and towards sesquiterpene production (Fig. 1). This can be achieved by decreasing activity of the FPP-consuming enzyme squalene synthase, either through engineered protein degradation [23] or transcriptional down-regulation [16, 26, 27]. These steps provide the basic principles of pathway optimization for sesquiterpene production in yeast.

An ideal microbial cell factory should simultaneously exhibit high specific production rate and high specific growth rate in a batch cultivation [28]. However, these two objectives are commonly incompatible due to the metabolic burden and/or metabolic imbalance found in the presence of engineered pathways [29–31]. An alternative option is to separate growth and production phases [31]. This can be achieved by induction of synthetic pathway genes upon an environmental stimulus occurring after sufficient biomass is accumulated [32].

Regulation of gene expression across a batch cultivation (expression pattern) is delicately controlled by the gene promoter, transcriptional regulatory networks and the environmental inputs (usually the cultivation conditions, including activator/repressor concentration in cultures) [33–36]. Native promoters and regulatory networks can readily be used for metabolic pathway construction and optimization if their response conditions are well characterized. Several promoters have been well characterized to achieve inducible gene expression systems in yeast; these include a copper-inducible promoter (*P*_*CUP1*_, induced by high concentration of copper ion), galactose-inducible promoters (the bi-directional *P*_*GAL1*_ and *P*_*GAL10*_ promoters, de-repressed in the absence of glucose and induced when galactose is present), a sucrose-inducible promoter (*P*_*SUC1*_, de-repressed in the absence of glucose and induced when sucrose is present), a high-affinity hexose transporter promoter (*P*_*HXT7*_, induced when glucose levels are low) and heat shock transcriptional factor Hsf1p-mediated promoters (*P*_*SSA1*_ and *P*_*HSP26*_) [33, 36–42]. In addition, sophisticated synthetic regulatory circuits have been designed, including circuits that respond to cell density via an engineered quorum-sensing system [32] and circuits that are activated by product feedback [43]. These promoters and regulatory networks can be further explored for optimizing gene expression regulation in metabolic engineering.

*Trans*-nerolidol is a sesquiterpene alcohol with applications as fragrance, flavour, precursor for synthetic vitamin E/K1 and others [23]. Previously, we engineered a *trans*-nerolidol production pathway in yeast in concert with MVA pathway augmentation and a protein-mediated flux down-regulation strategy at squalene synthase [23]. We achieved a titre of ~100 mg L^−1^, but observed a decreased growth rate when using constitutive overexpression of genes. To attain high-level production of nerolidol without a growth defect, further metabolic engineering is required. In this work, we engineered transcriptional regulation module that responds to bioprocess conditions to optimize growth and production for improved nerolidol titre, in combination with metabolic pathway optimization.

## Results

### Constitutive expression of genes results in decreased growth rates and constrains product titres

**Fig. 2:**
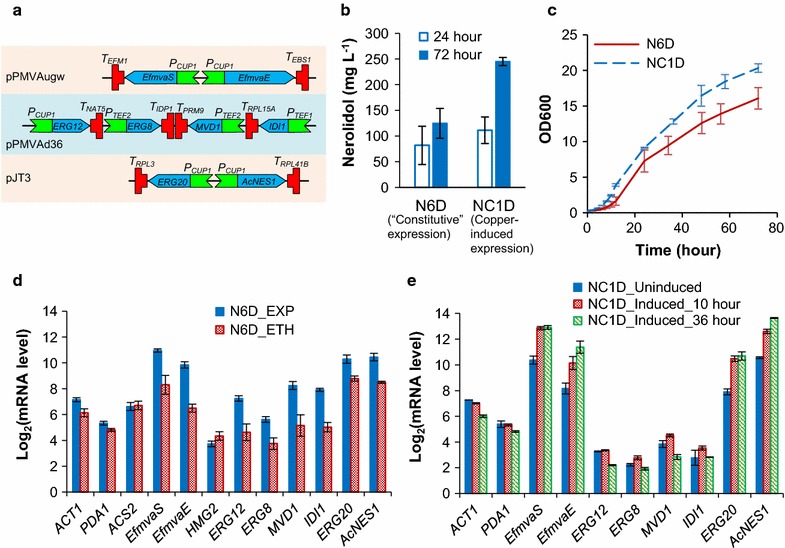
Characterizing strains N6D (with “constitutive” gene overexpression constructs) [23] and NC1D (with copper-inducible gene overexpression constructs): **a** the copper-inducible expression cassettes in plasmids pPMVAugw, pPMVAd36 and pJT3; **b** nerolidol titre at 24 and 72 h; **c** growth curves over batch cultivation; **d** mRNA levels in strain N6D in the exponential phase (EXP) and the ethanol phase (ETH, at 48 h); **e** mRNA levels in the strain NC1D in the pre-culture (without adding copper; un-induced) and in two-phase cultivation (with copper added; induced; at 10 or 36 h). Two-phase flask cultivation on 20 g L^−1^ glucose was employed; for the cultivation of NC1D, 100, 100 and 200 μM (final concentration) copper sulphate was added sequentially at 5, 10 and 24 h. mRNA levels were measured by quantitative real-time PCR. Mean values ± standard deviations are shown (*N* ≥ 3)

In our previous work, nerolidol production was improved by heterologously expressing more efficient upper MVA pathway genes from *Enterococcus faecalis* (*EfmvaS* and *EfmvaE*), overexpressing the yeast lower MVA pathway genes and FPP synthase and destabilizing squalene synthase (Erg9p) [23]. The resulting strain (Table 1) produced 104 ± 35 mg L^−1^ nerolidol over 72 h in batch cultivation on minimal medium with 20 g L^−1^ glucose. All of the genes, including nerolidol synthase (*AcNES1*) were overexpressed from plasmids using promoters with constitutive activity (*P*_*RPL4A*_ for *EfmvaS*, *P*_*RPL15A*_ for *EfmvaE*, *P*_*RPL8B*_ for *ERG12*, *P*_*SSB1*_ for *ERG8*, *P*_*RPL3*_ for *MVD1*, *P*_*YEF3*_ for *IDI1*, *P*_*TEF2*_ for *ERG20* and *P*_*TEF1*_ for *AcNES1*; Tables 1 and 2). These promoters exhibit high-level activities in the exponential phase when glucose is available, but dramatically decreased activities when glucose is depleted and cells have shifted to the ethanol growth phase [33]. Strain N6D exhibited half the specific growth rate (μ_max_ of 0.16 ± 0.03 h^−1^) of the wild-type CEN.PK reference strain (μmax of 0.30 ± 0.01 h^−1^) [23]. We presume that this is due to the metabolic burden from high expression levels of the heterologous synthetic pathway in the exponential phase. Furthermore, the decreased expression level after the diauxic shift might lead to low productivities in the post-exponential phase. To examine productivity across the fermentation period, 24- and 72-h samples were re-analysed using a new HPLC–UV method. Consistent with the GC–MS data obtained previously [23], a titre of 125 ± 30 mg L^−1^ was measured at 72 h. At 24 h, the titre was 82 ± 37 mg L^−1^ (Fig. 2b) and the productivity was calculated to 5.7 mg g^−1^ biomass h^−1^ in the exponential phase (from 0 to 24 h) compared to only 0.18 mg g^−1^ biomass h^−1^ in the post-exponential phase (from 24 to 72 h).

**Table 1:** *S. cerevisiae* strains used in this work

Strain	Genotype	Resource/reference
CEN.PK2-1C	*MATa ura3*-*52 trp1*-*289 leu2*-*3,112 his3Δ 1*	[44]
CEN.PK113-5D	*MATa ura3*-*52*	[44]
CEN.PK113-7D	*MATa*	[44]
*ILHA series strains*		
oH5	oURA3 derivative*; ERG9(1333, 1335)::yEGFP*-*CLN2* ^*PEST*^-*T*_*URA3*_-*loxP*-*KlURA3*-*loxP*	[23]
N6D	oH5 derivative; [pPMVAu8] [pPMVAd3] [pJT1]	[23]
NC1D	oH5 derivative; [pPMVAugw] [*TRP1*]^a^ [pJT3]	This work
o391	CEN.PK2-1C derivative; *HMG2* ^*K6R*^(*−152 ,−1*)*::HIS3*-*T*_*EFM1*_<*EfmvaS*<*P*_*GAL1*_–*P*_*GAL10*_>*ACS2*>*T*_*ACS2*_–*P*_*GAL2*_>*EfmvaE*>*T*_*EBS1*_ *–P*_*GAL7*_ *; pdc5* (−*31, 94*)*::P*_*GAL2*_>*ERG12*>*T*_*NAT5*_ *–P*_*TEF2*_>*ERG8*>*T*_*IDP1*_–*T*_*PRM9*_<*MVD1*<*P*_*ADH2*_ *−T*_*RPL15A*_<*IDI1*<*P*_*TEF1*_-*TRP1*	This work
N391DA	o391 derivative; *ERG9(1333, 1335)::CLN2* ^*PEST*^-*T*_*URA3*_-*loxP*-*KlURA3*-*loxP gal80::loxP*-*kanMX4*-*loxP* [pJT9R]	This work
oJ3	CEN.PK113-5D derivative; *gal80::loxP*-*kanMX4*-*loxP*	This work
GH4J3	oJ3 derivative; *ura3* (*1, 704*)*::KlURA3*	This work
G89J3	oJ3 derivative; *ura3* (*1, 704*)*::KlURA3*-*P*_*TEF1*_-*yEGFP*	This work
GB5J3	oJ3 derivative; *ura3* (*1, 704*)*::KlURA3*-*P*_*GAL1*_-*yEGFP*	This work
GB6J3	oJ3 derivative; *ura3* (*1, 704*)*::KlURA3*-*P*_*GAL10*_-*yEGFP*	This work
GQ3J3	oJ3 derivative; *ura3* (*1, 704*)*::KlURA3*-*P*_*GAL2*_-*yEGFP*	This work
GQ4J3	oJ3 derivative; *ura3* (*1, 704*)*::KlURA3*-*P*_*GAL7*_-*yEGFP*	This work

Symbol > or < indicates the direction of open reading frames

*a* the plasmid pPMVAd36 was transformed

**Table 2:** Plasmids used in this work

Plasmid	Features	Reference
pRS423	*E. coli/S. cerevisiae* shuttle plasmid; 2 μ, *HIS3*	[45]
pRS424	*E. coli/S. cerevisiae* shuttle plasmid; 2 μ, *TRP1*	[45]
pRS425	*E. coli/S. cerevisiae* shuttle plasmid; 2 μ, *LEU2*	[45]
pPMVAu8	pRS423: *P*_*RPL4A*_>*EfmvaS*>*T*_*EFM1*_–*P*_*RPL15A*_>*EfmvaE*>*T*_*EBS1*_	[23]
pPMVAd3	pRS424: *P*_*RPL8B*_>*ERG12*>*T*_*NAT5*_ *–P*_*SSB1*_>*ERG8*>*T*_*IDP1*_ *–P*_*RPL3*_>*MVD1*>*T*_*PRM9*_ *–P*_*YEF3*_>*IDI1*>*T*_*RPL15A*_	[23]
pJT1	pRS425: *P*_*TEF2*_>*ERG20*>*T*_*RPL3*_ *–P*_*TEF1*_ *–AcNES1*-*T*_*RPL41B*_	[23]
pPMVAugw	pRS423: *T*_*EFM1*_<*EfmvaS*<*P*_*CUP1*_ *–P*_*CUP1*_>*EfmvaE*>*T*_*EBS1*_	This work
pIMVAu1	pRS423: *HMG2* (−*309,* −*153*)-*HIS3*-*T*_*EFM1*_<*EfmvaS*<*P*_*GAL1*_ *–P*_*GAL10*_>*ACS2*>*T*_*ACS2*_ *–P*_*GAL2*_>*EfmvaE*>*T*_*EBS1*_–*P*_*GAL7*_>*HMG2* ^*K6R*^(*1, 292*)	This work
pPMVAd36	pRS424: *P*_*CUP1*_>*ERG12*>*T*_*NAT5*_–*P*_*TEF2*_>*ERG8*>*T*_*IDP1*_ *–T*_*PRM9*_<*MVD1*<*P*_*TEF2*_–*T*_*RPL15A*_<*IDI1*<*P*_*TEF1*_	This work
pIMVAd39T	pUC19: *pdc5* (−*277, −32*)-*P*_*GAL2*_>*ERG12*>*T*_*NAT5*_ *–P*_*TEF2*_>*ERG8*>*T*_*IDP1*_ *–T*_*PRM9*_<*MVD1*<*P*_*ADH2*_ *–T*_*RPL15A*_<*IDI1*<*P*_*TEF1*_-*TRP1*-*pdc5* (*95,373*)	This work
pJT3	pRS425: *T*_*RPL3*_<*ScERG20*<*P*_*CUP1*_–*P*_*CUP1*_>*AcNES1*>*T*_*RPL41B*_	This work
pJT9R	pRS425: *T*_*RPL3*_<*ScERG20*<*P*_*GAL1*_–*P*_*GAL2*_>*AcNES1*>*T*_*RPL41B*_	This work
pILGFP3	Yeast integration plasmid; *yEGFP* without promoter	[33]
pILGH4	pILGFP3: with *yEGFP* removed	[33]
pILGB5A	pILGFP3: *P*_*GAL1*_-*yEGFP*	[33]
pILGB6	pILGFP3: *P*_*GAL10*_-*yEGFP*	This work
pILGFPQ3	pILGFP3: *P*_*GAL2*_-*yEGFP*	This work
pITGFPQ4	pILGFP3: *P*_*GAL7*_-*yEGFP*	This work

Symbol > or < indicates the direction of open reading frame

The decreased nerolidol production rate may be attributed to the lower promoter activity we previously reported for these constitutive promoters during the ethanol phase [33]. Indeed, the mRNA levels of the genes for nerolidol production (*EfmvaS*, *EfmvaE*, *ERG12*, *ERG8*, *MVD1*, *IDI1*, *ERG20* and *AcNES1*) decreased 3- to 10-fold in the ethanol growth phase compared to the exponential growth phase (Fig. 2d). As noted previously [23], the mRNA levels of the reference genes *ACT1* and *PDC1* also decreased. Interestingly, in contrast to the classical ‘housekeeping’ genes *ACT1* and *PDC1,* acetyl-CoA synthase (*ACS2*) exhibited similar transcriptional levels in the exponential/ethanol growth phases.

### Copper-inducible expression improves nerolidol production

To avoid both the metabolic imbalances causing decreased growth rate in the exponential phase and the decreased productivities in the post-exponential phase, gene expression can be controlled using an induction system. The *CUP1* promoter is responsive to copper ion concentration [38]. In the absence of additional copper, the *CUP1* promoter exhibits a moderate expression level; with addition of high-concentration copper (above 100 μM), activity is induced to a level comparable to the strong *TEF1* promoter in exponential phase [33]. In contrast to the *TEF1* promoter, the *CUP1* promoter can maintain high expression activity in the ethanol phase (in the presence of 300 μM copper; [33]). These characteristics make the *CUP1* promoter an potentially useful candidate to address both of the aforementioned problems. To test the effect on sesquiterpene production, a new set of plasmids were constructed (Table 2): an *E. faecalis* upper MVA pathway plasmid (pPMVAgw) with *EfmvaS* and *EfmvaE* controlled by two divergent *CUP1* promoters; a yeast lower MVA pathway plasmid (pPMVAd36) with the mevalonate kinase gene *ERG12* controlled by the *CUP1* promoter and the other three genes (*ERG8*-phosphomevalonate kinase, *MVD1*-mevalonate pyrophosphate decarboxylase and IDI1-Isopentenyl diphosphate:dimethylallyl diphosphate isomerase) controlled by a *TEF1* promoter and two *TEF2* promoters, respectively; and finally, a nerolidol synthetic plasmid (pJT3) with *ERG20* and *AcNES1* controlled by two divergent *CUP1* promoters (Fig. 2a). The three plasmids were co-transformed into the Erg9p-destabilized strain oH5 [23] to generate strain NC1D (Table 1).

While the wild-type growth rate (μ_max_ = 0.30 ± 0.01 h^−1^) was not fully recovered, strain NC1D exhibited a 1.6-fold faster growth rate (μ_max_ = 0.25 ± 0.01 h^−1^) than strain N6D (Table 3), demonstrating that the metabolic imbalance in the exponential phase was partially relieved. Furthermore, higher nerolidol production was achieved, with a titre of 111 ± 26 mg L^−1^ at 24 h and 245 ± 8 mg L^−1^ at 72 h (Fig. 2b, c). This equates to production rates of 5.1 mg g^−1^ biomass h^−1^ in the period from 0 to 24 h, slightly lower than the rate in strain N6D; and 0.77 mg g^−1^ biomass h^−1^ in the post-exponential phase (from 24 to 72 h), fourfold higher than the rate in strain N6D (Table 3).

**Table 3:** The maximum growth rate (*μ*_max_) and nerolidol productivities of the engineered strain in flask cultivation on 20 g L^−1^ glucose

Strain	N6D	NC1D	N391DA
*μ*_max_ (h^−1^)	0.16 ± 0.03	0.25 ± 0.01	0.27 ± 0.01
*r*_nerolidol_ (0–24 h; mg g^−1^ biomass h^−1^)	5.73 ± 2.19	5.14 ± 1.29	1.40 ± 0.05
*r*_nerolidol_ (24–72 h; mg g^−1^ biomass h^−1^)	0.18 ± 0.08	0.77 ± 0.09	2.10 ± 0.02
Nerolidol titre (72 h; mg L^−1^)	124 ± 29	245 ± 8	393 ± 3
Nerolidol C-mole yield (%)	1.7 ± 0.4	3.3 ± 0.1	5.0 ± 0.3

Mean values ± standard deviations are shown (*N* ≥ 2)

To verify the relationship between gene expression levels and nerolidol production, the mRNA levels in strain NC1D were also analysed. Unexpectedly, the mRNA levels for four genes from the ‘lower’ MVA pathway (*ERG12*, *ERG8*, *MVD1* and *IDI1*; Fig. 2) were similar compared to those in the wild-type CEN.PK reference strain [23]. Furthermore, the overexpression cassettes could not be amplified from the genomic DNA (data not shown). The 2 μ plasmid originally containing the yeast lower MVA pathway was recovered from NC1D. Restriction mapping and DNA sequencing showed that DNA recombination occurred between pPMVAd36 and pJT3; the result of this recombination was a plasmid where the four lower MVA pathway gene expression cassettes from pPMVAd36 were replaced by the nerolidol synthase cassette from pJT3 (Additional file 1: Figure S1). This replacement pattern is consistent with two homologous recombination events occurring, one between the *CUP1* promoters for *ERG12* and *AcNES1*, and one between homologous sequences on the plasmid backbones. Despite this undesiriable recombination event, strain NC1D performed better than N6D (see above). Very low variability was observed between biological replicates for the mRNA levels (Fig. 2e) and nerolidol production (Fig. 2b), demonstrating that the plasmid recombination and resulting strain were stable.

Despite the loss of the four lower MVA pathway genes, the upper pathway genes *EfmvaS, EfmvaE* and *ERG20* and *AcNES1* controlled by *CUP1* promoters, were still present in NC1D. Consistent with our previous findings [33], the mRNA levels for these genes increased by about fourfold after copper induction, and high transcript levels were maintained in the ethanol growth phase for these genes (Fig. 2e). In the exponential phase under un-induced conditions, mRNA levels for *EfmvaS* and *EfmvaE* were 2 and sevenfold lower (Fig. 2e) than transcript levels driven by constitutive promoters (Fig. 2d). The ‘lower’ pathway was not augmented at all, considering the loss of the ‘lower’ MVA pathway genes during the recombination event. Together, these data support the idea that high transcription of MVA pathway genes during the exponential phase (driven by constitutive promoters) results in a metabolic imbalance that decreases growth rate and nerolidol titre. Applying the *CUP1* promoter to control synthetic pathway genes improved nerolidol production; however, plasmid recombination caused by repeated usage of the *CUP1* promoter is undesirable in a metabolic engineering context. Therefore, an alternative inducible expression system was developed.

### *Δgal80* enables auto-induction of *GAL* promoters over/after diauxic shift

**Fig. 3:**
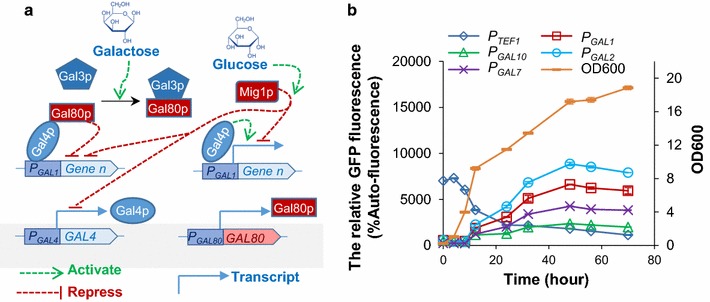
Schematic representing regulatory networks for the galactose-inducible promoter (*P*_*GAL1*_; **a**) and GFP fluorescence levels in *gal80Δ* background strains (**b**) with the *yEGFP* gene driven by *P*_*TEF1*_ (strain G89J3), *P*_*GAL1*_ (strain GB5J3), *P*_*GAL10*_ (strain GB6J3), *P*_*GAL2*_ (strain GQ3J3) and *P*_*GAL7*_ (strain GQ4J3). Cultures were grown on 20 g L^−1^ glucose. GFP fluorescence is expressed as percentage of exponential-phase auto-fluorescence of the reference strain (GH4J3). The growth curve (OD_600_) for GH4J3 is shown. Mean values ± standard deviations are shown (*N* = 2)

In a previous comparison of a set of commonly used promoters, the *GAL1* promoter drove the highest expression level [33]. The *GAL1* promoter and galactose-based cultivation have also been used in the initial laboratory development of several sesquiterpene-producing strains [46, 47]. However, it is not feasible to use galactose as a carbon source in industrial production due to its high cost. In the core galactose regulon, Gal80p binds the transcription activator Gal4p to inhibit Gal4p-mediated transcription initiation of the *GAL1* promoter in the absence of galactose; in the presence of galactose, transcriptional factor Gal3p binds with Gal80p to relieve Gal80p repression on Gal4p transcription activation (Fig. 3a) [48]. Galactose-independent (gratuitous) activation of the *GAL1* promoter can be achieved by disruption of the *gal80* repressor [49]. Additionally, galactose-inducible expression driven by *GAL1* promoter is co-regulated by Mig1p-mediated glucose-dependent repression: in the presence of glucose, Mig1p can bind to the *GAL1*, *GAL3* and *GAL4* promoters to inhibit gene transcription (Fig. 3a) [48, 50]. Mig1p-mediated repression can lead to the glucose-dependent repression of the *GAL1* promoter in a *gal80Δ* strain [16, 49, 51]. Consequently, it has been shown that the *GAL1* promoter can be automatically induced in a *gal80Δ* strain as the cells shift to the ethanol growth phase [51].

In the current study, to avoid repeated use of a single promoter for multiple genes, the promoters from the galactose metabolic genes (*GAL1*, *GAL10*, *GAL2* and *GAL7*) and a reference *TEF1* promoter were characterized in a *gal80Δ* strain (Tables 1, 2; Fig. 3b). GFP was used as a reporter to measure the promoter activities over an entire batch cultivation on 20 g L^−1^ glucose. Growth profiles were similar and showed a diauxic growth pattern in all strains (data for the *gal80Δ* control strain are shown in Fig. 3b; other data not shown). We showed previously that, under the same conditions, the diauxic shift pattern observed in the growth curve was coincident with glucose depletion and the start of ethanol consumption, and occurred at ~12 h [33]. Similar to our previous study in the wild-type strain [33], activity driven by the *TEF* promoter in the *gal80Δ* strain decreased dramatically during and after the diauxic shift. In contrast, the *GAL* promoters exhibited diauxie-inducible expression patterns: their activities started to increase at 12 h, peaking at 48 h. The strength of *GAL* promoters in the post-exponential phase was as follows: *P*_*GAL2*_>*P*_*GAL1*_>*P*_*GAL7*_>*P*_*GAL10*_. The auto-inducible expression pattern makes the *GAL* promoter in combination with *gal80Δ* very useful in strain development to simultaneously avoid the metabolic burden in the exponential phase and increase productivity in the ethanol growth phase.

### Auto-inducible *GAL* promoter regulation drives efficient nerolidol production

**Fig. 4:**
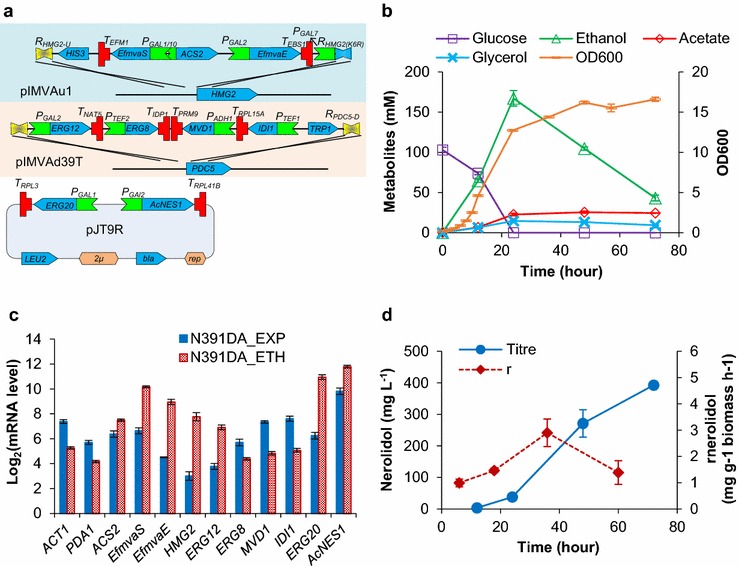
Characterizing strain N391DA with *gal80Δ*-*GAL* promoter constructs: **a** genetic modules/plasmid for the “*upper*” mevalonate pathway (pIMVAu1), the “*lower*” mevalonate pathway (pIMVAd39T) and the nerolidol synthetic genes (pJT9R); **b**, **d** metabolic and growth profiles (*N* = 2); **c** mRNA levels (*N* = 3) in the exponential phase (EXP) and the ethanol growth phase (ETH, at 36 h). Two-phase flask cultivation on 20 g L^−1^ glucose was employed. Mean values ± standard deviations are shown

To apply the modified galactose-inducible system (*GAL* promoters in *gal80Δ* background), a new nerolidol-producing strain, N391DA, was constructed (Fig. 4a; Tables 1, 2). In the “upper” MVA module, genes *EfmvaS* (HMG-CoA synthase), *ACS2* (native acetyl-CoA synthase) and *EfmvaE* (thiolase/HMG-CoA reductase) were controlled by *P*_*GAL1*_, *P*_*GAL10*_ and *P*_*GAL2*_, respectively. Overexpressing *ACS2* has previously been shown to increase the intracellular concentration of acetyl-CoA [21], which is the precursor metabolite for the MVA pathway; hence, it was included in the ‘upper’ module. The three expression cassettes were integrated into the *HMG2* (HMG-CoA reductase) promoter locus with the selection marker *HIS3*. At the same time, a *P*_*GAL7*_ promoter linked to a short sequence of the *HMG2* gene including a K6R mutation that stabilizes Hmg2p from degradation [52, 53] was introduced as a fusion with the native *HMG2*. As a result, *HMG2* ^*K6R*^ was expressed from the genome under the control of *P*_*GAL7*_. To construct a “lower” MVA module, *ERG12* (mevalonate kinase) was controlled by *P*_*GAL2*_; the other three genes (*ERG8*, *MVD1*, *IDI1*) were controlled by glucose-dependent “constitutive” promoters (*P*_*TEF2*_, *P*_*ADH1*_ and *P*_*TEF1*_, respectively). This construct was integrated with the *TRP1* selection marker into the *PDC5* locus, which encodes a weakly expressed pyruvate decarboxylase [54]. Dysfunction of *PDC5* does not cause a major change in yeast metabolism, because of complementation by isoforms *PDC1* and *PDC6* [55]. For the nerolidol synthesis module, *P*_*GAL1*_-controlled *ERG20* and *P*_*GAL2*_-controlled *AcNES1* were introduced on a 2μ plasmid. Finally, squalene synthase (Erg9p) was destabilized by the addition of an endoplasmic-reticulum mediated protein degradation sequence to reduce its ability to compete for FPP with nerolidol synthase [23], and *gal80* was disrupted to allow diauxic induction of *GAL* promoters. The resulting strain N391DA was evaluated through two-phase flask cultivation.

Strain N391DA exhibited a normal exponential growth with μmax of 0.27 ± 0.01 h^−1^—1.7-fold faster than the strain N6D and slightly faster than strain NC1D. Consistent with the yeast diauxic growth model, N391DA consumed glucose and produced ethanol in the exponential growth phase, and ethanol was subsequently consumed in the secondary growth phase (Fig. 4b). N391DA produced 38 mg L^−1^ nerolidol at 24 h, production rates of 1.4 mg g^−1^ biomass h^−1^ in the period from 0 to 24 h, fourfold lower than the rate in strain N6D. Nerolidol reached 392 ± 2 mg L^−1^ at 72 h, which translates to a production rate of 2.1 mg g^−1^ biomass h^−1^ in the post-exponential phase (from 24 to 72 h)—12-fold higher than the rate in strain N6D (Table 3). The 72 h titre represents a 60% improvement relative to NC1D with copper-inducible constructs. N391DA exhibited the highest specific nerolidol production rate (*r*_*nerolidol*_) between 24 and 48 h and 86% of nerolidol was produced after 24 h (Fig. 4d).

To verify the expression pattern of the genes controlled by the modified galactose-inducible system, the mRNA levels were analysed in strain N391DA (Fig. 4c). In the exponential phase, the expression levels of the reference genes *ACT1* and *PDA1* in N391DA were similar (two-tailed *t* test *p* > 0.1) to those in strains N6D and NC1D. As observed previously, their mRNA levels decreased significantly in the ethanol growth phase; this was also the case for mRNAs levels from the genes controlled by *TEF1*, *TEF2* and *ADH1* promoters. In contrast, the genes controlled by *GAL* promoters exhibited increased transcriptional levels: twofold for *ACS2*, 11-fold for *EfmvaS*, 22-fold for *EfmvaE*, 27-fold for *HMG2*, ninefold for *ERG12*, 26-fold for *ERG20* and fourfold for *AcNES1* in the ethanol growth phase, compared to in the exponential growth phase. These fold-changes are consistent with the expression pattern of the four *GAL* promoters as characterized above (Fig. 3).

### Sucrose as an alternative carbon source for nerolidol production

**Fig. 5:**
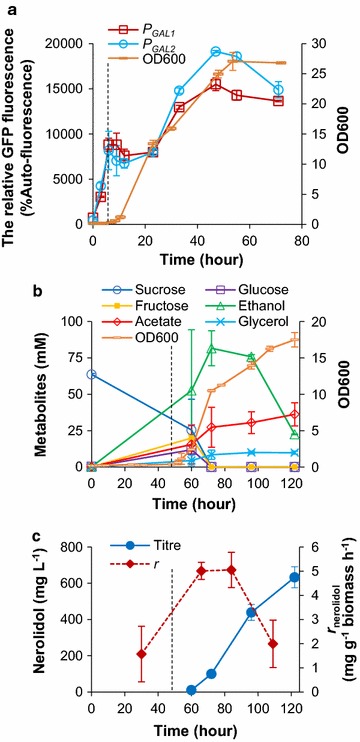
Effects of sucrose on *GAL* promoter activities in *gal80Δ* background and nerolidol production for strain N391DA: **a** the fluorescence levels of the *yEGFP* controlled by *GAL1* (strain GB5J3) or *GAL2* (strain GQ3J3) promoter over the batch cultivation on 20 g L^−1^ sucrose and the growth profile (OD600) of strain GH4J3; **b**, **c** metabolic and growth profile for strain N391DA in two-phase flask cultivation on 20 g L^−1^ sucrose. *Vertical dashed lines* indicate the end of lag phase. Mean values ± standard deviations are shown (*N* = 2)

Sucrose from sugar cane/sugar beet is an alternative carbon source to glucose in industrial fermentation [56–58]. As for the *GAL* genes, the invertase gene for sucrose utilization (*SUC2*) is under Mig1p-mediated glucose repression, which is relieved when yeast is cultivated on sucrose [59, 60]. Considering this, it is reasonable to expect that the expression output from *GAL* promoter in *gal80Δ* background strain might be different on sucrose than on glucose. To investigate the effect of sucrose on nerolidol production, *GAL* promoter activities in *gal80Δ* background strains and nerolidol production for strain N391DA were characterized on sucrose. Yeast strains were pre-cultured on 40 g L^−1^ glucose, which minimized *GAL* promoter activities in *gal80Δ* strains (data not shown). A 6-h lag phase was exhibited after transferring to 20 g L^−1^ sucrose medium; this lag phase was seen in both *gal80Δ* strains and the *GAL80* control strain (Fig. 5a; Additional file 1: Figure S2). During the lag phase on sucrose, glucose repression on *GAL* promoters was relieved. GFP expression driven by the *GAL1* and *GAL2* promoters increased sharply during the lag phase and plateaued during exponential growth (Fig. 5a); this expression level was similar to that observed in the ethanol growth phase of glucose batch cultivation (Fig. 3b). A further increase of ~twofold was observed after 24 h, presumably after the diauxic shift (Fig. 5a).

To characterize nerolidol production on sucrose, strain N391DA was first pre-cultured on 40 g L^−1^ glucose and then cultivated on 20 g L^−1^ sucrose. N391DA exhibited a 48-h lag phase on sucrose (Fig. 5b), dramatically longer than that for the GFP strains (Fig. 5a). The sugar profile demonstrated that strain N391DA first fermented sucrose and its hydrolysate products (glucose and fructose) into ethanol; the diauxic shift occurred by 72 h, and the strain then re-used ethanol in the post-exponential phase (Fig. 5b). In sucrose batch cultivation, the final nerolidol titre was 632 ± 57 mg L^−1^, 1.6-fold higher than in glucose batch cultivations. In addition, N391DA exhibited the highest post-exponential-specific nerolidol production rate of 5 mg g^−1^ biomass h^−1^ (Fig. 5c compared to Fig. 4d).

### Nerolidol production in fed-batch cultivation

To achieve high-titre nerolidol production for strain N391DA, fed-batch strategies were explored. We first explored a strategy designed to ensure that (a) fermentable sugars are catabolized through respiratory metabolism and (b) cultures are maintained under aerobic conditions. The initial feed rate was set to 1 mM glucose g^−1^ biomass h^−1^ with 600 g L^−1^ glucose feeding medium and then exponentially increased with a rate of 0.05 h^−1^; the feeding was switched off when dissolved oxygen (DO) was below 25% and maximum agitation and gassing were achieved, and the feeding was re-triggered when DO was above 30% (Additional file 1: Figure S3a). Two additional experiments were performed using volumetrically similar initial feed rates and feed solutions of 600 g L^−1^ sucrose and 400 g L^−1^ glucose/158 g L^−1^ ethanol, respectively.

**Fig. 6:**
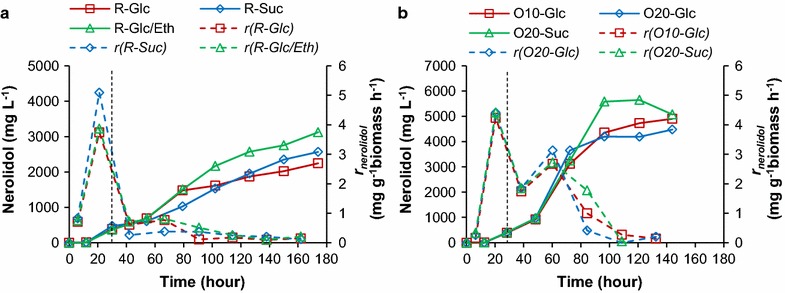
Nerolidol production for strain N391DA in fed-batch cultivations: **a** nerolidol production (*solid line*) and specific production rate (*r*; *dashed line*) in carbon-source-restricted DO-triggered fed-batch cultivation with feeding carbon source of 600 g L^−1^ glucose (R-Glc), or 600 g L^−1^ sucrose (R-Suc), or 400 g L^−1^ glucose 158 g L^−1^ ethanol mixture (R-Glc/Eth); **b** nerolidol production (*solid line*) and specific production rate (*r*; *dashed line*) in carbon-source-overflowed/carbon-source-pulsing fed-batch cultivation with feeding carbon source of 600 g L^−1^ glucose (with 10 g L^−1^ glucose pulse, O10-Glc; with 20 g L^−1^ glucose pulse, O20-Glc) or 600 g L^−1^ sucrose (with 20 g L^−1^ sucrose pulse, O20-Suc). *Vertical dashed line* approximately indicated the start of feeding. Growth and process values refer to Additional file 1: Figures S4, S5. *N* = 1

In the three experiments, the respiration quotients fluctuated around 1 for glucose or sucrose feeding processes, and around 0.9 for glucose/ethanol feeding process (Additional file 1: Figure S4), demonstrating that the fermentable sugars were catabolized through respiration. All processes began in batch mode using 20 g L^−1^ glucose as a carbon source and proceeded through diauxie and into the ethanol growth phase until DO started in increase sharply, triggering the feed. In this batch period, the three cultures produced 406 ± 57 mg L^−1^ nerolidol at 30 h (Fig. 6a). In the subsequent feeding phase, the glucose/ethanol feed provided higher nerolidol production than glucose or sucrose feeding; >2 g L^−1^ nerolidol was achieved at 102 h for glucose/ethanol and this titre was not achieved until 150 h for glucose and sucrose feeding. The final titre for the glucose/ethanol feed was >3 g L^−1^ at 174 h. For all three fed-batch experiments, the specific nerolidol production rates during feeding were lower than the rate observed in the ethanol growth phase in the batch process (Fig. 6a). The C-mole yield at 102 h was 2.0 ± 0.4% in these three fed-batches.

Next, the feeding strategy was altered with the aim to maintain overflow metabolism and cycling between ethanol production and consumption (Additional file 1: Figure S3b). After the batch phase, exponential feeding with an initial feeding rate of 3 mM glucose g^−1^ biomass h^−1^ for 600 g L^−1^ glucose (or volumetrically the same for 600 g L^−1^ sucrose) feeding medium and a specific increasing rate of 0.05 h^−1^ were applied. Once 50 g L^−1^ sugar had been fed, the feeding was paused to allow cells to consume the ethanol produced during sugar fermentation. Subsequently, 10 or 20 g L^−1^ sugar pulse feeding was repeatedly triggered by sharp DO increases (Additional file 1: Figure S5). The production of ethanol was confirmed by the respiration quotient being over 2 when the sugar was fed (Additional file 1: Figure S5). In the batch phase for the three batches, 404 ± 11 mg L^−1^ nerolidol was produced (Fig. 6b), consistent with the above results (Fig. 6a). In these three fed-batch processes (Fig. 6b), >4 g L^−1^ nerolidol was produced at 96 h, and the specific nerolidol production rates in the early feeding phase (t < 96 h) were noticeably higher than those in the carbon-restricted processes (Fig. 6a). At 96 h, the C-mole yield was 3.8 ± 0.1% in the two glucose-overflow fed-batches and 4.5% in the sucrose-overflowed fed-batch (Fig. 6b).

## Discussion

An efficient sesquiterpene-producing yeast platform is of broad industrial interest, because it can be applied for the production of various high-value sesquiterpenes as well as FPP-derived biofuels by simply substituting a specific terpenoid production pathway (Fig. 1). There are two key principles for increasing FPP availability for sesquiterpene production in yeast: enhancing the MVA pathway to increase precursor supply, and reducing squalene synthase activity to decrease its consumption of FPP [14, 17–19, 23, 26, 27, 51, 61]. This study aimed to couple the transcriptional regulation pattern of heterologously expressed pathway genes to bioprocess conditions to minimize metabolic imbalance and optimize heterologous sesquiterpene production.

In previous studies, constitutive promoters were most often used to control the expression of heterologous genes, whereas the optimization of expression strength over the different fermentation stages has not been well investigated [7, 8]. In the current study, three categories of transcription regulation patterns were applied to assemble synthetic pathways: constitutive, copper-inducible and diauxie-induced. Using reporter gene systems, we showed previously that expression outputs from constitutive promoters (including “classical” translational elongation factor promoters, *P*_*TEF1*_ and *P*_*TEF2*_; glycolytic promoter, *P*_*ADH1*_; ribosome biogenesis promoters, *P*_*RPL3*_, *P*_*RPL15A*_) dramatically decrease after the diauxic shift [33]. Our transcription data (Fig. 2d) and nerolidol production data (Fig. 2b**)** confirmed this. Nerolidol is not toxic and does not cause dramatic growth inhibition (Additional file 1: Figure S6), in contrast to monoterpenes [62]. But we also observed a decreased growth rate (Fig. 2c), which was consistent with metabolic burden from high-level expression in the exponential phase.

Using a copper-inducible promoter (*P*_*CUP1*_), gene expression was shifted from exponential to the ethanol growth phase (Fig. 2e). This resulted in improved growth rate during the exponential phase (Fig. 2c) and improved productivities after the diauxic shift (Fig. 2b). However, the repeated use of the *CUP1* promoter sequence led to undesirable homologous recombination and loss of introduced DNA fragments (Additional file 1: Figure S2). It is likely that similar losses have occurred previously when repeated promoter sequences have been used, for example, Beekwilder et al. [63] observed that almost half of colonies did not produce a carotenoid reporter product when they used the *TDH3* promoter to control several carotogenic pathway genes. Genetic instability at repeat sequences has been documented in yeast [64], and provides a challenge for large-scale modification/introduction of metabolic pathways due to limited availability of appropriate promoters [33].

To address this problem, we characterized four native *GAL* promoters (*P*_*GAL1*_, *P*_*GAL10*_, *P*_*GAL2*_ and *P*_*GAL7*_) in a *gal80Δ* genetic background to engineer a diauxie-inducible system [16, 65], and applied them to assemble the MVA pathway modules (Figs. 3, 4a). While these promoters in *gal80Δ* background have been used to control the expression of the genes from the MVA pathway and amorphadiene/isoprene synthetic pathway previously [16, 65], they have not been characterized in terms of behaviour throughout the fermentation nor have they been ranked. The relative strength of *P*_*GAL1*_ and *P*_*GAL10*_ has been examined previously with conflicting results. One study [41] observed *P*_*GAL10*_ to be stronger than *P*_*GAL1*_; in contrast, our study and two others [39, 66] showed *P*_*GAL1*_ to be stronger than *P*_*GAL10*_. It is possible that differences in experimental conditions caused this discrepancy. Using the *GAL* promoters, a further shift in gene expression from exponential to ethanol growth phase was achieved (Figs. 2e, 4c), which resulted in further improved growth during the exponential phase (Fig. 4b) and further improved productivities after the diauxic shift (Figs. 2b, 4d). Increased expression from the *GAL* promoters and improved nerolidol productivities were consistently observed when the engineered cells were cultivated on sucrose (Fig. 5). Together, these data demonstrate the importance of appropriate transcriptional control modules for optimized strain engineering. There are several genotypic differences between strains NC1D and N391DA, including promoter identity (*P*_*CUP1*_ vs. *P*_*GAL*_), gene location (plasmid vs. genome integration) and gene expression level; and we have not examined each in detail to determine relative contribution to the phenotype.

In a previous study using the *gal80Δ* background, the sesquiterpene amorphadiene was produced at >40 g L^−1^ titre in ethanol fed-batch cultivation, whereas it reached only ~3 g L^−1^ in glucose fed-batch cultivation [16]. Here, nerolidol production in fed-batch cultivation was also investigated in the *gal80Δ* background; in contrast to the previous study, two substrate-feeding strategies, sugar-restricted and sugar-surplus, were compared (Fig. 6). In the sugar-restricted feeding processes, sugar was expected to be catabolized through respiratory metabolism; in the sugar-surplus feeding processes, ethanol was expected to accumulate in the sugar respiro-fermentative metabolism after each sugar feed and to be re-consumed when sugar was depleted. The two feeding strategies resulted in similar growth profiles (comparing OD_600_ when t ≤ 102 or 96 h; Additional file 1: Figures S4, S5). However, in the carbon-overflow fed-batches, higher titre/yield/rate of nerolidol were achieved, compared to the substrate-restricted fed-batches (Fig. 6). In addition to different promoter behaviours throughout the fed-batch cultivation, the improvement in nerolidol production might be due to a MVA pathway response to “overflow” metabolism, as is seen in ethanol/acetate/glycerol production through glycolysis [67–69]. In support of this possibility, the concentrations of intracellular pyruvate, acetyl-CoA, acetoacetyl-CoA and FPP were shown to increase as the dilution rate increased in carbon-limited chemostat processes [70, 71]. The pyruvate node is a key metabolic node for regulation of carbon flux in yeast; specifically, it determines flux towards mitochondrial pyruvate dehydrogenase or the cytosolic pyruvate dehydrogenase bypass (and ultimately, acetyl-CoA, which provides substrate for the MVA pathway) [72, 73]. Moreover, flux distribution into the cytosolic pyruvate dehydrogenase bypass decreases with decreased glucose feeding rate [74]. This can explain the decreased flux through the MVA pathway for nerolidol production under the carbon-limited fed-batch.

## Conclusion

Here, we present an expanded modular genetic regulatory system to co-ordinate expression outputs with physiological behaviour of engineered cells under industrially relevant cultivation conditions. The gene transcriptional regulatory pattern for assembled metabolic pathways is critically important for metabolic functionality and optimal productivity in engineered strains. This principle should be considered for de novo assembly of heterogeneous metabolic pathways in the “design-build” construction cycle for industrial microbial producers. The diauxie-induction system, including the four characterized *GAL* promoters, is simple and efficient for developing a yeast strain for high-level sesquiterpene production. Further optimization to balance sugar and ethanol growth phases might significantly increase titres.

## Methods

### Plasmid and strain construction

Strains used in this work are listed in Table 1 and plasmids are listed in Table 2. Primers used in polymerase chain reaction (PCR) and details of PCR performed in this work are listed in Additional file 1: Table S1. Plasmid construction processes are listed in Additional file 1: Table S2.

Strain NC1D was generated by co-transforming plasmids pPMVAugw, pPMVAd36 and pJT3 into the Erg9p-destabilized strain oH5 (*ERG9*-*yEGFP*-*CLN2* ^*PEST*^) [23]. Strain o391D was generated by sequentially transforming strain CEN.PK2-1C [44] with *Pme*I-digested pIMVAd39T, *Swa*I-digested pIMVAu1 and an Erg9p-destabilization fragment (*ERG9* ^*C*-*terminal*^-*CLN2* ^*PEST*^-*T*_*URA3*_-*loxP*-*KlURA3*-*loxP*-*T*_*ERG9*_; Additional file 1: Table S1#21; [23]). The nerolidol production plasmid pJT9R was then transformed into strain o391D. Finally, *gal80* was disrupted by integrating a *LoxP*-*KanMX4*-*loxP* marker (Additional file 1: Table S1#23) to generate strain N391DA. N391DA was selected on SC-glutamate-high-glucose (SCGHG) agar plate with 300 μg mL^−1^ G-418. The nutrient recipe of the SCGHG agar is as follows: 1.6 g L^−1^ uracil-drop-out amino acid mixture [75], 1.7 g L^−1^ yeast nitrogen base (YNB) without ammonium sulphate (Sigma-Aldrich#Y1251), 1 g L^−1^ glutamate, 200 g L^−1^ glucose. For strains NC1D and N391DA, at least 3 independent colonies were stored separately in 20% glycerol at −80 °C for subsequent evaluation.

To disrupt *gal80*, a *LoxP*-*KanMX4*-*loxP* PCR fragment (Additional file 1: Table S1#23) was transformed into CEN.PK113-5D; the resultant strain oJ3 was transformed with *Swa*I-digested pILGH4, pILG89S, pILGB5AS, pILGB6S, pILGQ3 and pILGQ4 to generate strain GH4J3, GB5J3, GB6J3, GQ3J3 and GQ4J3, respectively.

### Two-phase flask cultivation and sampling

Nerolidol-producing strains were evaluated through two-phase flask cultivation using dodecane as a non-toxic organic extractant phase [76]. Synthetic minimal (SM) medium containing 6.7 g L^−1^ YNB (Sigma-Aldrich #Y0626; pH 6.0) with 20 g L^−1^ glucose as the carbon source was used (SM-glucose). 100 mM 2-(N-morpholino) ethanesulfonic acid (MES, Sigma-Aldrich#M8250) was used to buffer medium pH and the initial pH was adjusted to 6.0 by adding ammonium hydroxide. Strains were recovered from glycerol stocks by streaking on SM-glucose agar plates (for N6D and NC1D) or SCGHG agar plates (for N391DA) and pre-cultured in MES-buffered SM 20 g L^−1^ glucose (or 40 g L^−1^ glucose as indicated) medium to exponential phase (cell density OD_600_ between 1 and 4). Pre-cultured cells were collected by centrifugation and re-suspend in fresh media before initiating two-phase cultivations. Two-phase flask cultivation was initiated by inoculating pre-cultured cells to OD_600_ = 0.2 in 25 mL MES-buffered SM 20 g L^−1^ glucose medium (or 23 mL MES-buffered SM 20 g L^−1^ sucrose medium when cultures were not sampled for RNA extraction) in 250 mL flasks with solvent-resistant screw-caps [76]; 2 mL dodecane was added to extract nerolidol. Flask cultivation was performed at 30°C and 200 rpm. For all cultivations, about 3 mL culture was sampled before the end of exponential growth phase for OD measurement; meanwhile, dodecane and culture were sampled in 1:10 ratio for metabolite analysis (see below). For RNA extraction, 2 mL exponential-phase culture (OD_600_ = 1–1.5) and 1 mL ethanol growth-phase culture (at 36 or 48 h) were sampled, and cells were collected and stored at −80 °C.

### Fed-batch cultivation

Fed-batch cultivation was performed in DASGIP 400-mL fermenters (DASGIP#SR0400SS, Jülich, Germany). The medium for fed-batch cultivation was modified from previous reports [16, 77, 78]. 1× trace element composition and 1× vitamin composition from previous report [77] were used. Seed and batch media contained 15 g L^−1^ (NH4)_2_SO_4_, 8 g L^−1^ KH_2_PO_4_, 3 g L^−1^ MgSO_4_, 10× trace element composition and 10× vitamin composition; additionally, seed medium contained 40 g L^−1^ glucose, and batch medium contained 20 g L^−1^ glucose. Feed medium contained 9 g L^−1^ KH_2_PO_4_, 2.5 g L^−1^ MgSO_4_, 3.5 g L^−1^ K_2_SO_4_, 0.28 g L^−1^ Na_2_SO_4_, 10× trace element composition and 10× vitamin composition, with carbon source of 600 g L^−1^ glucose, or 600 g L^−1^ sucrose, or a mixture of 400 g L^−1^ glucose and 158 g L^−1^ ethanol. Ammonium hydroxide (~10 M) was used to adjust pH value to 5. Dissolved oxygen (DO) was monitored using a PreSens minisensor oxygen metre (PreSens, Germany) and controlled with a proportional–integral (PI; DO set-point, 30%; *P*, 0.5; Ti, 50 s; *PI* output, 0–100%) agitation-gassing-cascade (PI input 0–60% ~agitation output 300–600 rpm; *PI* input 20–60% ~air gassing output 1.58–3.16 L h^−1^) controller (DASGIP control). Off-gas was dried using a 10 °C chiller and 50 mL self-indicating silica gel was analysed using the DASGIP off-gas analyser.

In batch cultivation, 130 mL batch medium and 25 ml dodecane were used initially; seed culture in exponential phase (OD_600_ = 5–10) was directly inoculated to OD_600_ = 0.2 in batch culture. Medium feeding was triggered when the DO increased sharply after carbon sources in batch medium were depleted. Medium feeding was programmed using the DASGIP VB.NET script feeding controller (for feeding script logic charts refer to Additional file 1: Figure S3).

### Quantitation of mRNA level

Total RNA was extracted using a yeast RiboPure™ RNA Purification Kit (Ambion #AM1926) or a TRIzol® Plus RNA Purification Kit (Ambion #12183555). After DNase treatment, 0.1 or 0.2 μg total RNA was used for first-strand cDNA synthesis in a 10 μL reaction using ProtoScript® II Reverse Transcriptase (NEB #M0368). The diluted cDNA was used as the template for quantitative real-time (qRT) PCR (primers are listed in Additional file 1: Table S1#29 to #40). KAPA SYBR® FAST qPCR Kit (Kapa Biosystems#KP-KK4601) and CFX96 Touch™ Real-Time PCR Detection System (BIO-RAD) were used in qRT-PCR. Yeast genomic or plasmid DNA was used to prepare the standard curve. *C*_t_ values were analysed using CFX Manager Software (Bio-Rad Laboratories, QLD Australia). The mRNA levels (N pg^−1^ total RNA) were calculated by referring to the standard curve equations.

### GFP fluorescence assay

To monitor GFP fluorescence over the entire batch cultivation, cells were cultivated aerobically in MES-buffered 20 mL SM-glucose (SM-sucrose) medium in 100 mL flasks. Samples were taken at indicated time points, and GFP fluorescence in single cells was analysed immediately after sampling using a flow cytometer (BD Accuri™ C6; BD Biosciences, USA). Cultures were diluted after 12 h by 10 volume water for flow cytometer analysis. GFP fluorescence was excited by a 488 nm laser and monitored through a 530/20 nm band-pass filter (FL1.A); 10,000 events were counted per sample. The GFP fluorescence signal (FL1.A) was corrected according to the values of FSC.A (forward scatter detector) and SSC.A (side scatter detector) [33]. GFP fluorescence level was expressed as the percentage relative to the average background auto-fluorescence from exponential-phase cells of a GFP-negative reference strain GH4J3.

### Metabolite analysis

Extracellular metabolites were analysed by the Metabolomics Australia Queensland Node. Trans-nerolidol was analysed using a novel HPLC method. Dodecane samples (in some cases, diluted with dodecane) were diluted in 40-fold volume of ethanol. Ethanol-diluted dodecane samples of 20 μL were injected into a Zorbax Extend C18 column (4.6 × 150 mm, 3.5 µm, Agilent PN: 763953-902) with a guard column (SecurityGuard Gemini C18, Phenomenex PN: AJO-7597). Analytes were eluted at 35 °C at 0.9 mL/min using the mixture of solvent A (high purity water, 18.2 kΩ) and solvent B (45% acetonitrile, 45% methanol and 10% water), with a linear gradient of 5–100% solvent B from 0 to 24 min, then 100% from 24 to 30 min and finally 5% from 30.1 to 35 min. Analytes of interest were monitored using a diode array detector (Agilent DAD SL, G1315C) at 196 and 202 nm wavelengths. Spectral scans were also performed on each of the compounds from 190 to 400 nm in steps of 2 nm to confirm their identity and purity. Trans-nerolidol primary pharmaceutical reference standard (93.7% purity; Sigma-Aldrich #04610590) was used to prepare the standard curve for quantification.

Glucose, ethanol, acetate, glycerol and mevalonate were analysed through ion-exclusion chromatography [79]. Ion-exclusion chromatography was performed using an Agilent 1200 HPLC system and an Agilent Hi-Plex H column (300 × 7.7 mm, PL1170-6830) with guard column (SecurityGuard Carbo-H, Phenomenex PN: AJO-4490). Analytes were eluted isocratically with 4 mM H_2_SO_4_ at 0.6 mL/min at 65 °C. Glucose, ethanol, glycerol and mevalonate were monitored using a refractive index detector (Agilent RID, G1362A); acetate and mevalonate were detected using an ultraviolet–visible light absorbance detector (Agilent MWD, G1365B) at 210 nm. For sucrose, glucose and fructose analysis, analytes were eluted isocratically using high purity water (18.2 MΩ cm) as the mobile phase, at 0.4 mL/min for 21 min, and sugars (sucrose, glucose and fructose) were monitored using a refractive index detector (Agilent RID, G1362A).

### Physiological feature calculation

Physiological parameters were calculated as reported previously [80]: the maximum growth rates are the linear regression coefficients of the ln OD_600_ versus time during the exponential growth phase; one unit of OD_600_ equals 0.23 g L^−1^ biomass; the specific production rate of nerolidol (*r*_nerolidol_, mg g^−1^ biomass h^−1^) was calculated by dividing Δ nerolidol titre (mg L^−1^) with the integral of biomass (g L^−1^) in defined time (h).

## Additional file

**Additional file 1: Table S1.** Primers and PCR fragments amplified/used in this work. PXXX, promoter of gene *XXX; T*_*XXX*_, terminator of gene XXX; Y-GDNA, CEN.PK113-7D genomic DNA; sequence annealing to template in primers is shown in red and italics; over-lap sequence for over-lap extension PCR and Gibson Assembly is underlined; restriction sites used in cloning are shown in **bold**. **Table S2.** Molecular construction of plasmids used in this work. **Figure S1.** Plasmid rearrangement in the strain NC1D. Plasmid pPMVAd36 and [TRP1] from NC1D were digested by restriction enzymes *Not*I, *Sal*I, *Sph*I, *BamH*I and *Sbf*I and gel figure was shown as the right-bottom figure. **Figure S2.** The growth profile of strain GH4 [CEN.PK113-5D derivative; *ura3(1, 704 )::KlURA3*] (1) on sucrose. The cells were pre-cultured on 40 g L^-1^ glucose. Mean values from duplicate experiments are shown. **Figure S3.** Logic charts for fed-batch feeding scripts: (a) carbon-source-restricted/DO-triggered fed-batch cultivation; (b) carbon-source-overflowed/carbon-source-pulsing fed-batch cultivation. F_s_, feeding flow storage value; DO_t_, dissolved oxygen on-line value at time t; DOL, lowest dissolved oxygen storage value; T1, storage time; t, on-line time; μ, specific rate of feeding flow increasement; N, agitatation speed; Nmax, the maxium agitation speed; FV_s_, feeding volume storage value; FV_t_, feeding volume on-line value; V_t_, culture volume on-line value. **Figure S4.** Growth (OD600) and process values (Dissovled oxygen, DO; oxygen transfer rate, OTR; carbon transfer rate; CTR; respiration quotient, RQ) in fed-batch cultivation for strain N391DA, with feeding logics in Fig. S1a employed. (a&b), 600 g L^-1^ glucose feeding; (c&d), 600 g L^-1^ sucrose feeding; (e&f), 400 g L^-1^ glucose and 158 g L^-1^ ethanol feeding. **Figure S5.** Growth (OD600) and process values (Dissovled oxygen, DO; oxygen transfer rate, OTR; carbon transfer rate; CTR; respiration quotient, RQ) in fed-batch cultivation for strain N391DA, with feeding logics in Fig. S1b employed. (a&b), 600 g L^-1^ glucose feeding with 10 g L^-1^ glucose pulse; (c&d), 600 g L^-1^ glucose feeding with 20 g L^-1^ glucose pulse; (e&f), 600 g L^-1^ sucrose feeding with 20 g L^-1^ sucrose pulse. **Figure S6.** The influence of nerolidol on yeast growth. Synthetic minimal medium was used, which contained 6.7 g L^-1^ yeast nitrogen base (Sigma-Aldrich #Y0626; pH 6.0) and 20 g L^-1^ glucose. Isomer-mixed nerolidol (Sigma-Aldrich #H59605) was used. Tween 80 was added to homogenize nerolidol into liquid medium. Mean values ± standard deviations are shown (N = 3).

## Abbreviations

IPP: isopentenyl pyrophosphate
DMAPP: dimethylallyl pyrophosphate
FPP: farnesyl pyrophosphate
MVA: mevalonate
*P*_*XXXX*_: promoter for gene XXXX
