# Supplementary material for: Coupling gene regulatory patterns to bioprocess conditions to optimize synthetic metabolic modules for improved sesquiterpene production in yeast
Source: Biotechnol Biofuels. 2017 Feb 21;10:43. doi: 10.1186/s13068-017-0728-x (PMC5320780; doi:10.1186/s13068-017-0728-x)
Supplement: Supplementary file 1 — Additional file 1: Table S1. Primers and PCR fragments amplified/used in this work. PXXX, promoter of gene XXX; T XXX, terminator of gene XXX; Y-GDNA, CEN.PK113-7D genomic DNA; sequence annealing to template in primers is shown in red and italics; over-lap sequence for over-lap extension PCR and Gibson Assembly is underlined; restriction sites used in cloning are shown in bold. Table S2. Molecular construction of plasmids used in this work. Figure S1. Plasmid rearrangement in the strain NC1D. Plasmid pPMVAd36 and [TRP1] from NC1D were digested by restriction enzymes NotI, SalI, SphI, BamHI and SbfI and gel figure was shown as the right-bottom figure. Figure S2. The growth profile of strain GH4 [CEN.PK113-5D derivative; ura3(1, 704 )::KlURA3] (1) on sucrose. The cells were pre-cultured on 40 g L-1 glucose. Mean values from duplicate experiments are shown. Figure S3. Logic charts for fed-batch feeding scripts: (a) carbon-source-restricted/DO-triggered fed-batch cultivation; (b) carbon-source-overflowed/carbon-source-pulsing fed-batch cultivation. Fs, feeding flow storage value; DOt, dissolved oxygen on-line value at time t; DOL, lowest dissolved oxygen storage value; T1, storage time; t, on-line time; μ, specific rate of feeding flow increasement; N, agitatation speed; Nmax, the maxium agitation speed; FVs, feeding volume storage value; FVt, feeding volume on-line value; Vt, culture volume on-line value. Figure S4. Growth (OD600) and process values (Dissovled oxygen, DO; oxygen transfer rate, OTR; carbon transfer rate; CTR; respiration quotient, RQ) in fed-batch cultivation for strain N391DA, with feeding logics in Fig. S1a employed. (a&b), 600 g L-1 glucose feeding; (c&d), 600 g L-1 sucrose feeding; (e&f), 400 g L-1 glucose and 158 g L-1 ethanol feeding. Figure S5. Growth (OD600) and process values (Dissovled oxygen, DO; oxygen transfer rate, OTR; carbon transfer rate; CTR; respiration quotient, RQ) in fed-batch cultivation for strain N391DA, with feeding logics in F [file 13068_2017_728_MOESM1_ESM.docx]

# Appendix A

**Table S1:** Primers and PCR fragments amplified/used in this work. *P_XXX_*, promoter of gene XXX; *T_XXX_*, terminator of gene *XXX*; Y-GDNA, CEN.PK113-7D genomic DNA; sequence annealing to template in primers is shown in red and italics; over-lap sequence for over-lap extension PCR and Gibson Assembly is underlined; restriction sites used in cloning are shown in **bold**.

| No. | PCR fragment | Over-lap extension PCR fragment | Primer | Sequence (5’ -> 3’) |
| --- | --- | --- | --- | --- |
| 1 | *EfmvaS*- *T_EFM1_* from pPMVAu8 | *P_CUP1_*- *EfmvaS*- *T_EFM1_* | PM4pRS423s | TAAATCAGCTCATTTTTTAACCAATAGGCCGAAAT*CGGCAAAATCCCTTATAAGGATCCA* |
|  |  |  | PPM8+EFM1tas | CGGCAAAATCCCTTATAAGGATCCAGGTACC*TACTGAAATTCATGACACAAATAAC* |
|  |  |  | PPM8+EfmvaSaa | GAATTCAAA*ATGACCATCGGTATTGACAAAATCTCC* |
|  | *P_CUP1_* from Y-GDNA |  | PPM8+TEF1pas | AAAAGGAGATTTT*GTCAATACCGATGGTCATTTTG* |
|  |  |  | PPM8+CUP1pa-s | GTCAATACCGATGGTCATTTTGAATTC*TTTATGTGATGATTGATTGATTGATTG* |
|  |  |  | PPM8+CUP1pa-a | CAATTCCTTAAGCTTATCTATCCTCGAG*TAAGCCGATCCCATTACCGACATT* |
|  |  |  | PPM8+MCSa | GCATGCCTTACGACTTAGTCGA*CAATTCCTTAAGCTTATCTATCC* |
| 2 | *P_CUP1_* from Y-GDNA | *P_CUP1_*-*EfmvaE*- *T_EBS1_* | PPM8+MCSs | CTCGAGGATAGATAAGCTTAAGGA*ATTGTCGACTAAGTCGTAAGG* |
|  |  |  | PPM8+CUP1pss | ATTGTCGACTAAGTCGTAAGGCATGC*TAAGCCGATCCCATTACCGACATT* |
|  |  |  | PPM8+CUP1ps-a | CTATTATTACTACAGTCTTCATTTTACTAG*TTTTATGTGATGATTGATTGATTGATTG* |
|  |  |  | PPM8+TEF1psa | TCAATGCGT*CTATTATTACTACAGTCTTCATTTTAC* |
| 3 | *EfmvaE*- *T_EBS1_* from pPMVAu8 |  | PPM8+EfmvaEss | ACTAGTAAA*ATGAAGACTGTAGTAATAATAGACGCATTGAG* |
|  |  |  | PM4pRS423a | ACGCCAAGCGCGCAATTAACC*CTCACTAAAGGGAACAAAAGCTGGA* |
| 4 | *P_GAL1/10_* from Y-DNA | *P_GAL1/10_-ACS2-P_GAL2_* | PMsuGAL1-10ps | TCAATACCGATGGTCATTTTGAATTC*TATAGTTTTTTCTCCTTGACGTTA* |
|  |  |  | PMsuGAL1-10pa | TTATGTTCCTTGATTGTCATTTTGTCGAC*TTATATTGAATTTTCAAAAATTCTTACTT* |
|  | *ACS2* from Y-DNA |  | PMsuACS2s | TTGAAAATTCAATATAAGTCGACAAA*ATGACAATCAAGGAACATAAAGTAG* |
|  |  |  | PMsuACS2a | GTAAACCTCCTTGGATTAGTACAGCATG*CATTACTTTATTCCCACTATAGG* |
|  | *P_GAL2_* from Y-DNA |  | PMsuGAL2ps | AGTGGGAATAAAGTAATGCATGC*TGTACTAATCCAAGGAGGTTTACG* |
|  |  |  | PMsuGAL2pa | CTATTATTACTACAGTCTTCATTTTACTAGT*TATGAAAGAATTATTTTTTTTATTATGTT* |
| 5 | *HMG2*-U [*HMG2*(-309, -153)] from Y-DNA |  | PIM1HMG2ps | TTTTAACCAATAGGCCGAAATCGGCAAAATCCCTTATTT*AAATAGACCATTGATCGTTAG* |
|  |  |  | PIM1HMG2pa | TCATTCTGAACGAGGCGCGCTTTC*TGTGGTAGGTTCTTGAGGTGATTTAG* |
| 6 | *HIS3* from Y-DNA |  | PIM1HIS3s | TCAGAAGGCCGTTTTCTAAATCACCTCAAGAACCTACCACA*GAAAGCGCGCCTCGTTCAG* |
|  |  |  | PIM1HIS3a | ATGTCGTTATTTGTGTCATGAATTTCAGTAGGATC*CTAGTACACTCTATATTTTTTTATG* |
| 7 | *P_GAL7_* from Y-DNA |  | PIM1GAL7ps | TTCTTTGGATCAGATACAAAATCTGGTTTGAACGCGAGCTC*TTTGCCAGCTTACTATCCT* |
|  |  |  | PIM1GAL7pa | TATCGTTCTTAAGGGAAGTGACATTTT*TGAGGGAATATTCAACTGTTTTT* |
| 8 | *HMG2^K6R^*(1, 292) from Y-DNA |  | PIM1HMG2K6As | AAAACAGTTGAATATTCCCTCAAAA*ATGTCACTTCCCTTAAGAACGATAG* |
|  |  |  | PIM1HMG2K6Aa | GGTTCCTGGCCTTTTGCTGGCCTTTTGCTCACATGTATTT*AAATATCGGCGGCTTCTTTA* |
| 9 | *PDC5*  (-277, -32) from Y-GDNA | *PDC5*(-277, -32)-*HIS3*- *PDC5*  (95, 373) | PI3PDC5Us | TAAAACGACGGCCAGTGAATTCGTTT*AAACTAATACGTAAACCTGCATTAAGGT* |
|  |  |  | PI3PDC5Ua | ACTCCTAGCGCGGCCGCTTTCCTGCAGG*CTCTTTGAGATTGATTATGTGAAATAAGTGT* |
|  | *HIS3* from pRS423 |  | PI3HIS3s | ATCAATCTCAAAGAGCCTGCAGGAAAGCGGCCGC*GCTAGGAGTCACTGCCAGGTA* |
|  |  |  | PI3HIS3a | TCATAAAGCTTATCCAAAAGAGATTT*AAATGAGCTGATTTAACAAAAATTTAACG* |
|  | *PDC5*  (95, 373) from Y-GDNA |  | PI3PDC5Ds | GTTAAATTTTTGTTAAATCAGCTCATTTAAAT*CTCTTTTGGATAAGCTTTATGAAGTC* |
|  |  |  | PI3PDC5Da | ATGATTACGCCAAGCTTGCATGCGTTT*AAACAGTGAAGTCACCGTTACC* |
| 10 | *P_TEF1_* from Y-GDNA | *P_TEF1_*-*ERG12*-*T_NAT5_* | PM32TEF1ps | GCAAAATCCCTTATAA**CCTGCAGG***GTATAAACAATGCATACTTTGTACGT* |
|  |  |  | PM32TEF1pa | GCAGAAGTTAAGAACGGTAATGACAT*TTTGTAATTAAAACTTAGATTAGATTGC* |
|  | *ERG12*-*T_NAT5_* from pPMVAd3 |  | PM32ERG12s | AATCTAAGTTTTAATTACAAA*ATGTCATTACCGTTCTTAACTTCTGC* |
|  |  |  | PM3NAT5ta | As above |
| 11 | *P_TEF2_* from Y-GDNA | *P_TEF2_*- *ERG8*- *T_IDP1_* | PM32TEF2ps | AGAATCACGAAGAGCT**GAGCTC***ACTTTGTTATGTAGAGTTTTTTTAGC* |
|  |  |  | PM32TEF2pa | CTGAAGGCTCTCAACTCTGACAT*GTTTAGTTAATTATAGTTCGTTGAC* |
|  | *ScERG8*- *T_IDP1_* from pPMVAd3 |  | PM32ERG8s | GTCAACGAACTATAATTAACTAAAC*ATGTCAGAGTTGAGAGCCTTCAG* |
|  |  |  | PM3IDP1ta | As above |
| 12 | *TRP1* from pRS423 |  | PI3ToTRP1ps | GTGGTGGCACTACCAGCGGCCGC*GTTAACGACATTACTATATATATAATATAGG* |
|  |  |  | PI3ToTRP1pa | TCATAAAGCTTATCCAAAAGAGATTT*AAATGAGCTGATTTAACAAAAATTTAACGC* |
| 13 | *P_TEF2_* from Y-GDNA | *P_TEF2_*- *MVD1*- *T_IDP1_* | PPM3+TEF2ps1 | CTTACATAGGTGGTGGCACTACCAGTCGAC*ACTTTGTTATGTAGAGTTTTTTTAGCTACC* |
|  |  |  | PPM3+TEF2pa | GATGCTGTGTAAACGGT*CATGTTTAGTTAATTATAGTTCGTTG* |
|  | *MVD1*-*T_PRM9_* from pPMVAd3 |  | PPM3+MVD1s | CAACGAACTATAATTAACTAAAC*ATGACCGTTTACACAGCATC* |
|  |  |  | PPM3+MVD1a1 | GCTGTTCTAATTCGTCCAAAAGTTACTAGT*TGTTTCAATTACGCAGGACCCTTTTACGAT* |
| 14 | *P_TEF1_* from Y-GDNA | *P_TEF1_*- *IDI1*- *T_RPL15A_* | PPM3+TEF1ps | AAA**GCGGCCGCG***TATAAACAATGCATACTTTGTACGTTC* |
|  |  |  | PPM3+TEF1pa | ATACTATTGTTGTCGGCAGT*CATTTTGTAATTAAAACTTAGATTAGATTG* |
|  | *ScIDI1*- *T_RPL15A_* from pPMVAd3 |  | PPM3+IDI1s | CAATCTAATCTAAGTTTTAATTACAAA*ATGACTGCCGACAACAATAGTAT* |
|  |  |  | PPM3+IDI1a | AAA**GTCGAC***TGGTAGTGCCACCACCTATGTAA* |
| 15 | *P_CUP1_* from Y-GDNA | *P_CUP1_*-*ERG12*-*T_NAT5_* | PM36CUP1ps | TAA**CCTGCAGG***TAAGCCGATCCCATTACCGACATT* |
|  |  |  | PM36CUP1pa | GCAGAAGTTAAGAACGGTAATGACAT*TTTATGTGATGATTGATTGATTG* |
|  | *ERG12*-*T_NAT5_* from pPMVAd3 |  | PM36ERG12s | CAATCAATCAATCATCACATAAA*ATGTCATTACCGTTCTTAACTTCTGC* |
|  |  |  | PM3NAT5ta | CCGTGTGTACTCCTCTG**GAGCTC***TTCGTGATTCTCCTGCAGTGGAAC* |
| 16 | *P_GAL2_* from Y-GDNA | *P_GAL2_-ERG12-T_NAT5_* | PMd38GAL2ps | ATAA**CCTGCAGG***TGTACTAATCCAAGGAGGTTTACG* |
|  |  |  | PMd38GAL2pa | GCAGAAGTTAAGAACGGTAATGA*CATTATGAAAGAATTATTTTTTTTATTATGTT* |
|  | *ERG12*-*T_NAT5_* from pPMVAd3 |  | PMd38ERG12s | AACATAATAAAAAAAATAATTCTTTCATA*ATGTCATTACCGTTCTTAACTTCTGC* |
|  |  |  | PM3NAT5ta | As above |
| 17 | *P_ADH1_* from Y-GDNA | *P_ADH1_-MVD1-T_IDP1_* | PPM39ADH1ps | CTTACATAGGTGGTGGCACTACCAGTCGAC*GCTCTTTTCCGATTTTTTTCTAAAC* |
|  |  |  | PPM39ADH1pa | CGGATGCTGTGTAAACGGT*CATTGTATATGAGATAGTTGATTGTATGCT* |
|  | *MVD1*-*T_PRM9_* from pPMVAd3 |  | PPM39MVD1s | AGCATACAATCAACTATCTCATATACA*ATGACCGTTTACACAGCATCCGT* |
|  |  |  | PPM3+MVD1a1 | As above |
| 18 | *T_RPL3_* from Y-GDNA | *P_CUP1_*- *ScERG20*- *T_RPL3_* | PJT1pRS425s | GGCCAGTGAGCGCGCGTAATACGACTCACTATAG*GGCGAATTGGGTACCGGG* |
|  |  |  | PJT+PRL3aa | GGGCGAATTGGGTACCGGGCCC*ATTGTAGCAAAGATTGTAAGGAAATAGCA* |
|  |  |  | PJT+PRL3as | AAGCAAATAATAGTGACCTGCAG*GAAGTTTTGTTAGAAAATAAATCATTTTTTAATTGAG* |
|  | *ERG20* from Y-GDNA |  | PJT+ERG20s | ATTTATTTTCTAACAAAACTTCCTGCAGG*TCACTATTATTTGCTTCTCTTGTAAACTTTG* |
|  |  |  | PJT+ERG20a | AATCAATCATCACATAAAGCTAGCAAA*ATGGCTTCAGAAAAAGAAATTAGGA* |
|  | *P_CUP1_* from Y-GDNA |  | PJT+CUP1pa-s | CTTTTTCTGAAGCCATTTTGCTAGC*TTTATGTGATGATTGATTGATTGATTG* |
|  |  |  | PPM8+CUP1pa-a | As above |
|  |  |  | PPM8+MCSa | As above |
| 19 | *P_CUP1_* from Y-GDNA | *P_CUP1_*-*AcNES1*- *T_RPL4A1B_* | PPM8+MCSs | As above |
|  |  |  | PPM8+CUP1pss | As above |
|  |  |  | PJT+CUP1ps-a | CTGCTGCGGTAGCCATTTTGGATCC*TTTATGTGATGATTGATTGATTGATTG* |
|  | *AcNES1* (Artificially synthesized ) |  | PJT+AcNES1s | CAATCATCACATAAAGGATCCAAA*ATGGCTACCGCAGCAGGTCCTATC* |
|  |  |  | PJT+AcNES1a | CTCTCAATCCGCCATGGTCACTA*TTATAAAGATGTGTTATAGATCATTGCCT* |
|  | *T_RPL41B_* from Y-GDNA |  | PJT+RPL41Bs | TAACACATCTTTATAATAGTGACCATG*GCGGATTGAGAGCAAATCGTTAAGT* |
|  |  |  | PJT1RPL41Bta | TGGAGCTCCACCGCGG*ACGCCACAGAAACCTCAGAATAAGCTA* |
|  |  |  | PJT1pRS425a | CAAGCGCGCAATTAACCCTCACTAAAGGGAACAAAAGC*TGGAGCTCCACCGCGGACG* |
| 20 | *P_GAL1_* from Y-GDNA | *P_GAL1_-ald6-P_GAL2_* | PJT8GAL1-10ps | TAATTTCTTTTTCTGAAGCCATTTTGCTAGC*TATAGTTTTTTCTCCTTGACGTTA* |
|  |  |  | PJT8GAL1-10pa | TGTCAAAGTGTAGCTTAGTCATTTTACTAGT*TTATATTGAATTTTCAAAAATTCTTACTT* |
|  | Partial *ald6* from Y-GDNA |  | PJT9Isos | AGTAAGAATTTTTGAAAATTCAATATAAACTAGTAAA*GCCCTATACTTTGCTTCTTTATG* |
|  |  |  | PJT8ALD6a | GTAAACCTCCTTGGATTAGTACACTCGAG*GTTAGTTTTCTTTGGATATAGCAGT* |
|  | *P_GAL2_* from Y-GDNA |  | PJT8GAL2ps | CTATATCCAAAGAAAACTAACCTCGAG*TGTACTAATCCAAGGAGGTTTACG* |
|  |  |  | PJT8GAL2pa | GCTGCGGTAGCCATTTTGGATCC*TATGAAAGAATTATTTTTTTTATTATGTT* |
| 21 | *ERG9*^C-terminal^-*CLN2*^PEST^-*T_URA3_*-*LoxP*-*KlURA3*-*LoxP*-*T_ERG9_* |  | PPGErg9-Cln2s | TTATTATATATACACTTTACACAGAGCGGCTGTAGGTTCT*GGATCCTCTAAAGGTGAAGA* |
|  |  |  | PRSERG9Cra | AGGATAGGCCTCTACCTATTATGTAAGTACTTAGTT*ATTGTTCGGAGTTGTTTGTTTATG* |
| 22 | Verify *ERG9* c-terminal modification | | PQvERG9s | AGAAATGTACCAGGATAAATTACCTCC |
|  |  |  | PQvERG9a | AACAGGACGGCGAAACTTTAATACG |
| 23 | *LoxP-KanMX4-loxP* from pUG6 |  | PdGAL80s | CAGCGTATACAATCTCGATAGTTGGTTTCCCGTTCTTTC*CAGCTGAAGCTTCGTACGCTG* |
|  |  |  | PdGAL80a | TATAAACGCTCTCGATTAACCTGTGTAATATCAGAGCAT*CATAGGCCACTAGTGGATCTG* |
| 24 | Verify *gal80* disruption | | PVdGAL80s | ACTGCTGGTCCTTGCCGAC |
|  |  | | PVdGAL80a | CCAAGCACAGGGCAAGATGC |
| 25 | *P_GAL10_* from Y-GDNA | | PPGGAL10ps | TTT**GTCGAC***TATAGTTTTTTCTCCTTGACGTTAAAG* |
|  |  | | PPGGAL10pa | TTT**GGATCC***CATTTATATTGAATTTTCAAAAATTCTTACTT* |
| 26 | *P_GAL2_* from Y-GDNA | | PPGGAL2ps | AAA**GTCGAC***TGTACTAATCCAAGGAGGTTTAC* |
|  |  | | PPGGAL2pa | AAA**GGATCC***CATTATGAAAGAATTATTTTTTTTATTATGTTAATC* |
| 27 | *P_GAL7_* from Y-GDNA | | PPGGAL7ps | AAA**GTCGAC***TTTGCCAGCTTACTATCCTTC* |
|  |  | | PPGGAL7pa | AAA**GGATCC***CATTTTTGAGGGAATATTCAACTG* |
| 28 | Verify *ura3* locus modification | | PPGura3Fs | ATGTGGCTGTGGTTTCAGGGTC |
|  |  |  | PPGura3Fa | TTCAATGCGTCCATCTTTACAGTCCT |
| 29 | PRTACT1s |  |  | ATGCAAACCGCTGCTCAA |
|  | PRTACT1a |  |  | AGTTTGGTCAATACCGGCAGA |
| 30 | PRTPDA1s |  |  | TTGATTGATCTAGGTATTGCCACTG |
|  | PRTPDA1a |  |  | AATTTGGCTTCTGGAGGAGGA |
| 31 | PRTACS2s |  |  | CTCCATACACCAAAGTTCAATCTG |
|  | PRTACS2a |  |  | CAAAGCTGGCTTGTCGGGATTAG |
| 32 | PRTHMG2s |  |  | AGTTCCCAGGACGAGCCTATC |
|  | PRTHMG2a |  |  | GGCAGTTTACCGTTGACGACA |
| 33 | PRTERG12s |  |  | GCGGTTGCTCTTTGACTTTGT |
|  | PRTERG12a |  |  | GTCCCACCCAAGTCTGTTTCA |
| 34 | PRTERG8s |  |  | AAACTAGCTTATTGGATGATTGCC |
|  | PRTERG8a |  |  | TAGCGGTTTGAGCCCTAAGAT |
| 35 | PRTMVD1s |  |  | CAGCTTGAGGCTTTCAACCAT |
|  | PRTMVD1a |  |  | TGTGGGCCTGAACCGACTT |
| 36 | PRTIDI1s |  |  | GTTAGAGACTTCAAATGGGTTTC |
|  | PRTIDI1a |  |  | GTCATCTAATTGCTCCCACCAG |
| 37 | PRTERG20s |  |  | TTCCGCAGAACAAAGAAAGAC |
|  | PRTERG20a |  |  | GGCAATAGACTCTTCATATTCGTG |
| 38 | PRTEfmvaEs |  |  | CTGCTTCATGTCACGCTTTCG |
|  | PRTEfmvaEa |  |  | CTAAGGCCAATGGAACGGATA |
| 39 | PRTEfmvaSs |  |  | GAATCCATCATATACAGTAGAAGAG |
|  | PRTEfmvaSa |  |  | CAACTGCACCTGAACCATAAGAA |
| 40 | PRTAcNES1s |  |  | TCAGCCAAAGATGAAGACCAAG |
|  | PRTAcNES1a |  |  | TCAAACATTTCCATGCTTCGG |

**Table S2:** Molecular construction of plasmids used in this work.

| Plasmid | Construction process |
| --- | --- |
| pPMVAugw | *P_CUP1_*-*EfmvaS*-*T_EFM1_*, *P_CUP1_*-*EfmvaE*-*T_EBS1_* and *Psi*I/*Sac*I-digested pRS423 were co-transformed into the *trp1*-deleted CEN.113-7D strain to assemble pPMVAugw. The plasmid was recovered by transforming total yeast genomic DNA into *E.coli*. |
| pIMVAu1 | *P_GAL1/10_-ACS2-P_GAL2_* was cloned into *Spe*I/*EcoR*I sites of pPMVAugw to generate pPMVAusu; *HMG2*-U, *HIS3, P_GAL7_, HMG2^K6R^*(1, 292), and *Bam*HI/*Sac*I-digested pPMVAusu were co-transformed into the *his3-*deleted CEN.113-7D to assemble pIMVAu1. The plasmid was recovered by transforming total yeast genomic DNA into *E.coli*. |
| pPMVAd36 | *PDC5*(-277, -32)-*HIS3*- *PDC5*(95, 373) was cloned into *Sph*I/*Eco*RI pUC19 to generate the plasmid pIToPDC5. The larger fragment from *Not*I/*Sbf*I digestion of pPMVAd3 was cloned into *Not*I/*Sbf*I sites of pIToPDC5 to generate pIMVAd3. *Sbf*I/*Sac*I-digested *P_TEF1_*-*ERG12*-*T_NAT5_* was cloned into *Sbf*I/*Sac*I sites in pIMVAd3 to generate pIMVAd31. *Sac*I/*Spe*I-digested *P_TEF2_*- *ERG8*- *T_IDP1_* was cloned into *Sac*I/*Spe*I sites of pIMVAd31 to generate pIMVAd32. *P_RPL3_*- *MVD1*- *T_IDP1_* was cloned into *Spe*I/*Sal*I sites of pIMVAd32 through *in-vitro* Gibson assembly to generate pIMVAd33. *TRP1* from pRS424 was cloned into *Not*I/*Swa*I sites of pIMVAd33 through in-vitro Gibson assembly to generate pIMVAd33T. *Not*I/*Sal*I-digested *P_TEF1_*- *IDI1*- *T_RPL15A_* was cloned into *Not*I/*Sal*I sites of pIMVAd33T to generate pIMVAd34T. *P_TEF2_*- *MVD1*- *T_PRM9_* was cloned into SpeI/SalI sites of pIMVAd34T through *in-vitro* Gibson assembly to generate pIMVAd35T. The larger fragment of *Not*I/*Sbf*I digestion of pIMVAd35T was ligated with the smaller fragments of *Not*I/*Sbf*I digestion of pPMVAd3 to generate pPMVAd35. *Sbf*I/*Sac*I-digested *P_CUP1_*-*ERG12*-*T_NAT5_* was cloned into *Sbf*I/*Sac*I sites in pIMVAd35T to generate pIMVAd36T.The larger fragment from *Not*I/*Sbf*I digestion of pIMVAd36T was ligated with the smaller fragments of *Not*I/*Sbf*I digestion of pPMVAd3 to generate pPMVAd36. |
| pIMVAd39T | *P_GAL2_-ERG12-T_NAT5_* was cloned into *Sbf*I/*Sac*I sites of pIMVAd35T to generate pIMVAd38T. *P_ADH1_-MVD1-T_PRM9_* was cloned into SpeI/SalI sites of pIMVA38T through *in-vitro* Gibson assembly to generate pIMVAd39T. |
| pJT3 | *P_CUP1_*- *ERG20*-*T_RPL3_*, *P_CUP1_*-*AcNES1*-*T_RPL41B_* and *Sac*I/*Apa*I-digested pRS425 were co-transformed into CEN.PK113-16B to assemble pJT3. The plasmid was recovered by transforming total yeast genomic DNA into *E.coli*. |
| pJT9R | *P_GAL1_-ald6-P_GAL2_* was cloned into *BamH*I/*Nhe*I sites of pJT3 through *in-vitro* Gibson assembly to generate pJT9R. |
| pILGFPB6S | *P_GAL10_* was cloned into *Xho*I/*BamH*I sites of pILGFP3 (1) to generate pILGFPB6S. |
| pILGFPQ3S | *P_GAL2_* was cloned into *Xho*I/*BamH*I sites of pILGFP3 (1) to generate pILGFPQ3S. |
| pILGFPQ4S | *P_GAL7_* was cloned into *Xho*I/*BamH*I sites of pILGFP3 (1) to generate pILGFPQ4S. |


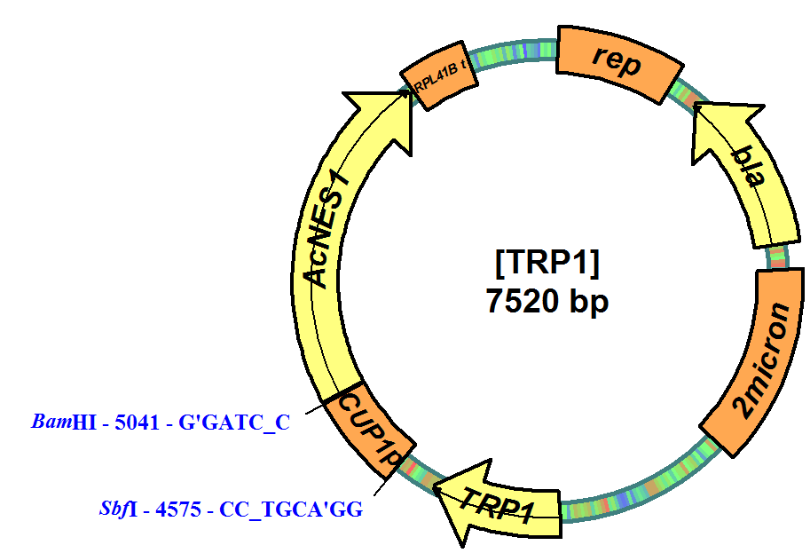

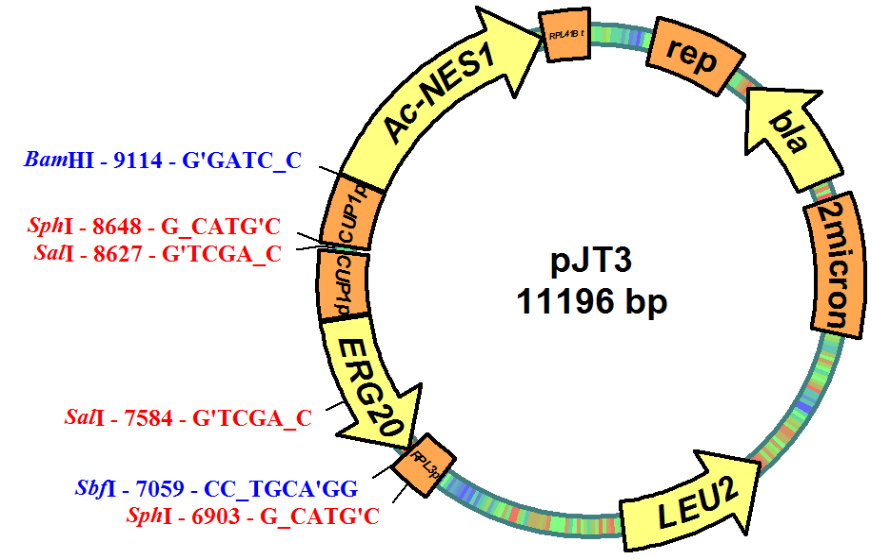

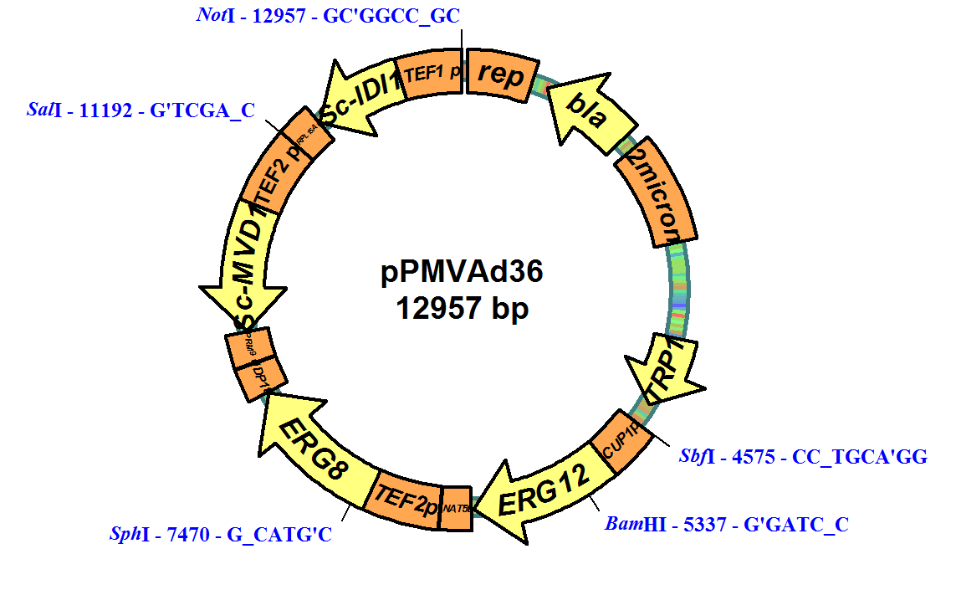


Confirmed by DNA sequencing

Plasmid rearrangement


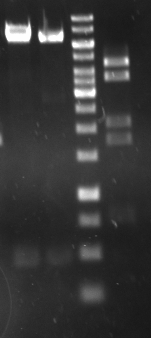


1 2 M 3

Lane 1 & 2, digested *TRP1* plasmids recovered from NC1D; Lane M, Thermo Scientific GeneRuler 1kb DNA Ladder (10000, 8000, 6000, 5000, 4000, 3500, 3000, 2500, 2000, 1500, 1000, 750, 500, 250 bp from top to bottom); Lane 3, digested pPMVAd36

Fig. S1 Plasmid rearrangement in the strain NC1D. Plasmid pPMVAd36 and [TRP1] from NC1D were digested by restriction enzymes *Not*I, *Sal*I, *Sph*I, *Bam*HI and *Sbf*I and gel figure was shown as the right-bottom figure.

Fig. S2 The growth profile of strain GH4 [CEN.PK113-5D derivative; *ura3(1, 704 )::KlURA3*] (1) on sucrose. The cells were pre-cultured on 40 g L^-1^ glucose. Mean values from duplicate experiments are shown.

Fig. S3 Logic charts for fed-batch feeding scripts: (a) carbon-source-restricted/DO-triggered fed-batch cultivation; (b) carbon-source-overflowed/carbon-source-pulsing fed-batch cultivation. F_s_, feeding flow storage value; DO*_t_*, dissolved oxygen on-line value at time t; DO_L_, lowest dissolved oxygen storage value; T1, storage time; t, on-line time; μ, specific rate of feeding flow increasement; N, agitatation speed; N_max_, the maxium agitation speed; FV_s_, feeding volume storage value; FV*_t_*, feeding volume on-line value; V_t_, culture volume on-line value.


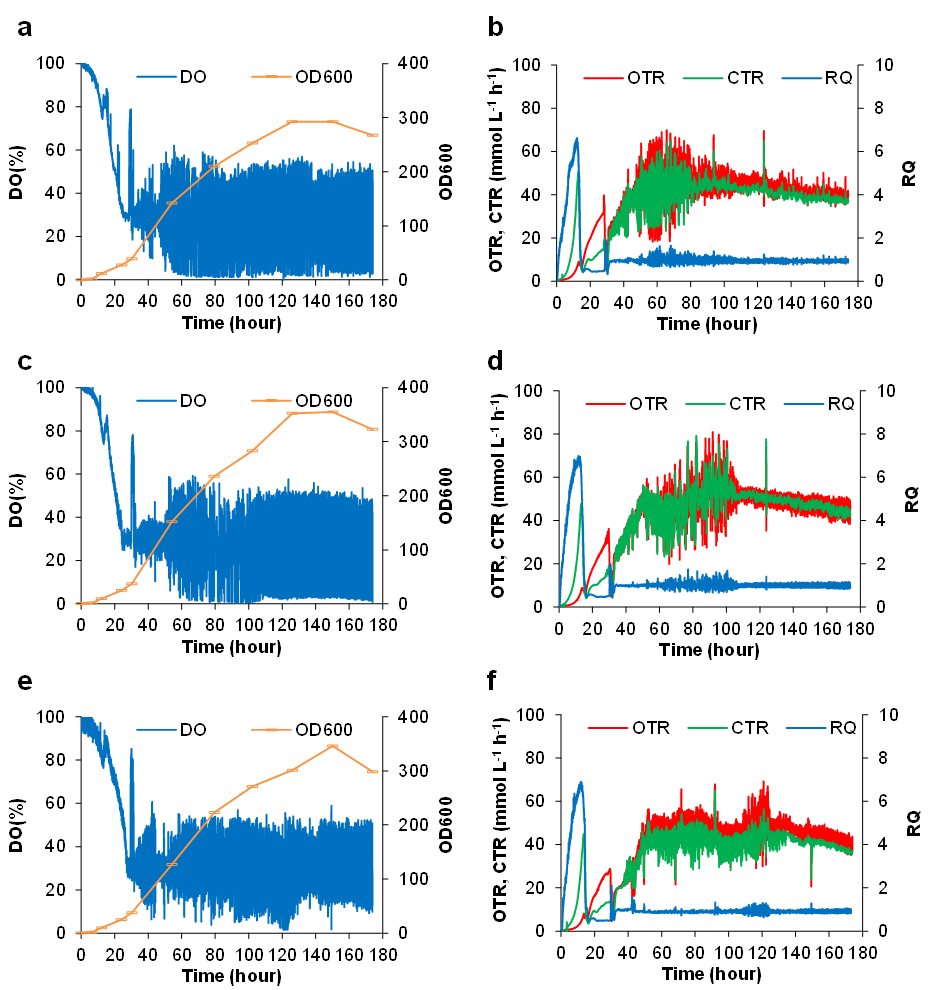


Fig. S4 Growth (OD600) and process values (Dissovled oxygen, DO; oxygen transfer rate, OTR; carbon transfer rate; CTR; respiration quotient, RQ) in fed-batch cultivation for strain N391DA, with feeding logics in Fig. S1a employed. (a&b), 600 g L^-1^ glucose feeding; (c&d), 600 g L^-1^ sucrose feeding; (e&f), 400 g L^-1^ glucose and 158 g L^-1^ ethanol feeding.


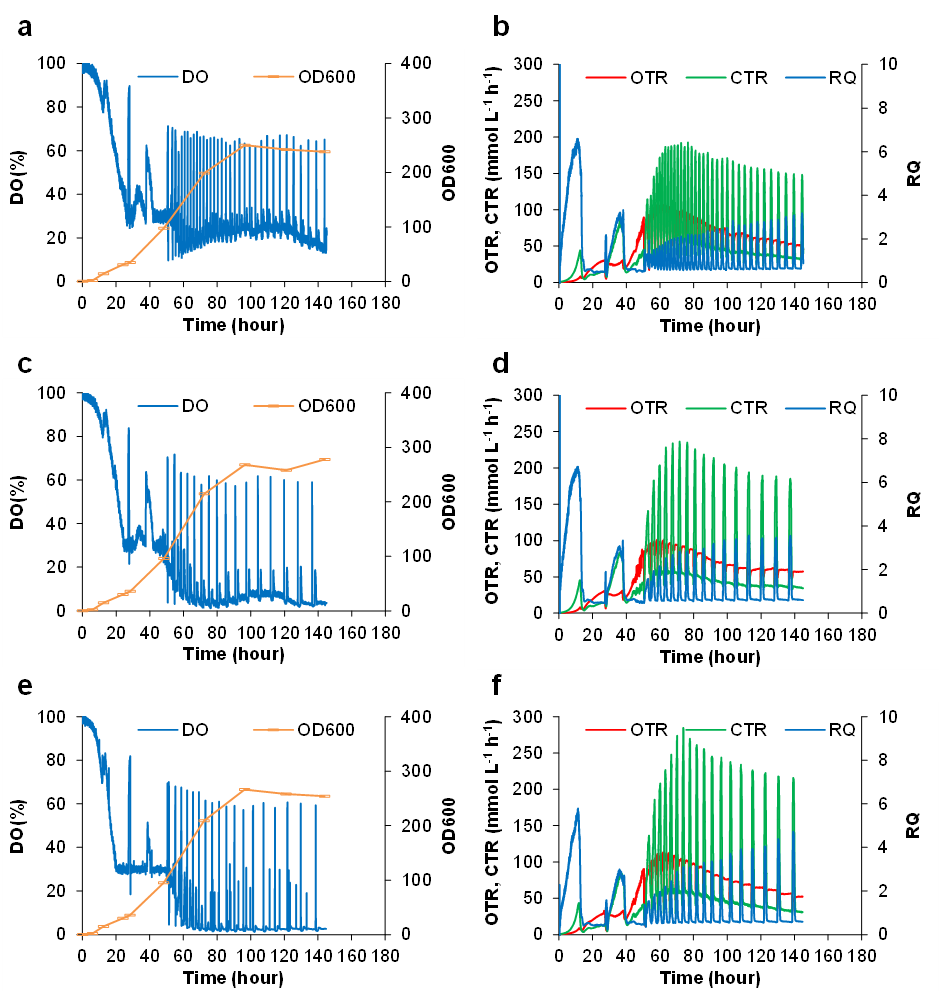


Fig. S5 Growth (OD600) and process values (Dissovled oxygen, DO; oxygen transfer rate, OTR; carbon transfer rate; CTR; respiration quotient, RQ) in fed-batch cultivation for strain N391DA, with feeding logics in Fig. S1b employed. (a&b), 600 g L^-1^ glucose feeding with 10 g L^-1^ glucose pulse; (c&d), 600 g L^-1^ glucose feeding with 20 g L^-1^ glucose pulse; (e&f), 600 g L^-1^ sucrose feeding with 20 g L^-1^ sucrose pulse.


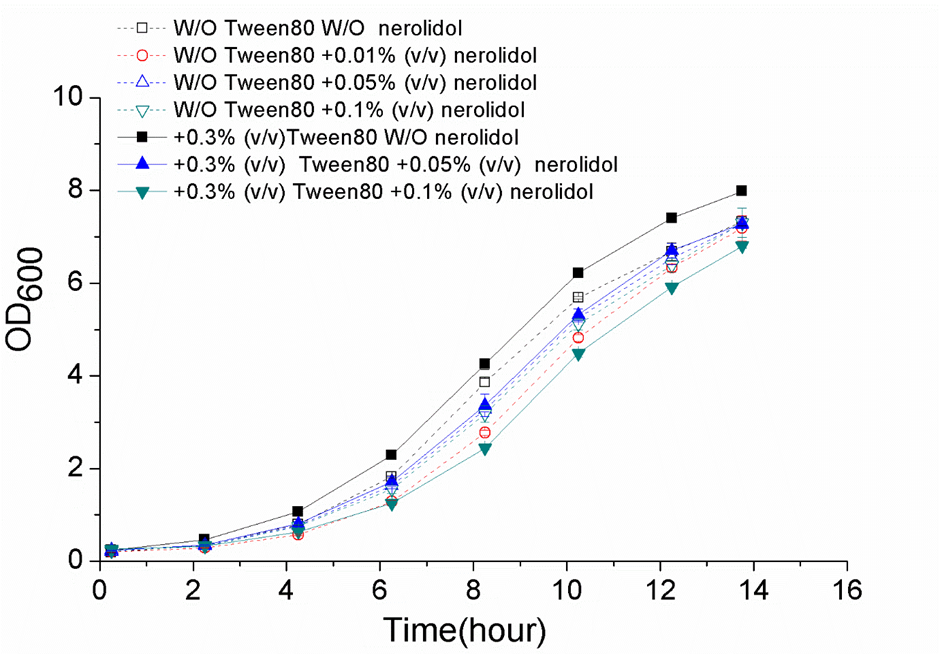


Fig. S6 The influence of nerolidol on yeast growth. Synthetic minimal medium was used, which contained 6.7 g L^-1^ yeast nitrogen base (Sigma-Aldrich #Y0626; pH 6.0) and 20 g L^-1^ glucose. Isomer-mixed nerolidol (Sigma-Aldrich #H59605) was used. Tween 80 was added to homogenize nerolidol into liquid medium. Mean values ± standard deviations are shown (N = 3).

Reference

1. Peng, B., Williams, T., Henry, M., Nielsen, L. and Vickers, C. (2015) Controlling heterologous gene expression in yeast cell factories on different carbon substrates and across the diauxic shift: a comparison of yeast promoter activities. *Microbial cell factories*, **14**, 91.
